# Lightweight and mobile artificial intelligence and immersive technologies in aviation

**DOI:** 10.1186/s42492-025-00203-z

**Published:** 2025-09-03

**Authors:** Graham Wild, Aziida Nanyonga, Anam Iqbal, Shehar Bano, Alexander Somerville, Luke Pollock

**Affiliations:** 1 School of Science, University of New South Wales, Canberra, ACT 2612 Australia; 2 School of Engineering and Technology, University of New South Wales, Canberra, ACT 2612 Australia

**Keywords:** Artificial intelligence, Aviation, Augmented reality, Mobile computing

## Abstract

This review examines the current applications, benefits, challenges, and future potential of artificial intelligence (AI) and immersive aviation technologies. AI has been applied across various domains, including flight operations, air traffic control, maintenance, and ground handling. AI enhances aviation safety by enabling pilot assistance systems, mitigating human error, streamlining safety management systems, and aiding in accident analysis. Lightweight AI models are crucial for mobile applications in aviation, particularly for resource-constrained environments such as drones. Hardware considerations involve trade-offs between energy-efficient field-programmable gate arrays and power-consuming graphics processing units. Battery and thermal management are critical for mobile device applications. Although AI integration has numerous benefits, including enhanced safety, improved efficiency, and reduced environmental impact, it also presents challenges. Addressing algorithmic bias, ensuring cybersecurity, and managing the relationship between human operators and AI systems are crucial. The future of aviation will likely involve even more sophisticated AI algorithms, advanced hardware, and increased integration of AI with augmented reality and virtual reality, creating new possibilities for training and operations, and ultimately leading to a safer, more efficient, and more sustainable aviation industry.

## Introduction

**Fig. 1:**
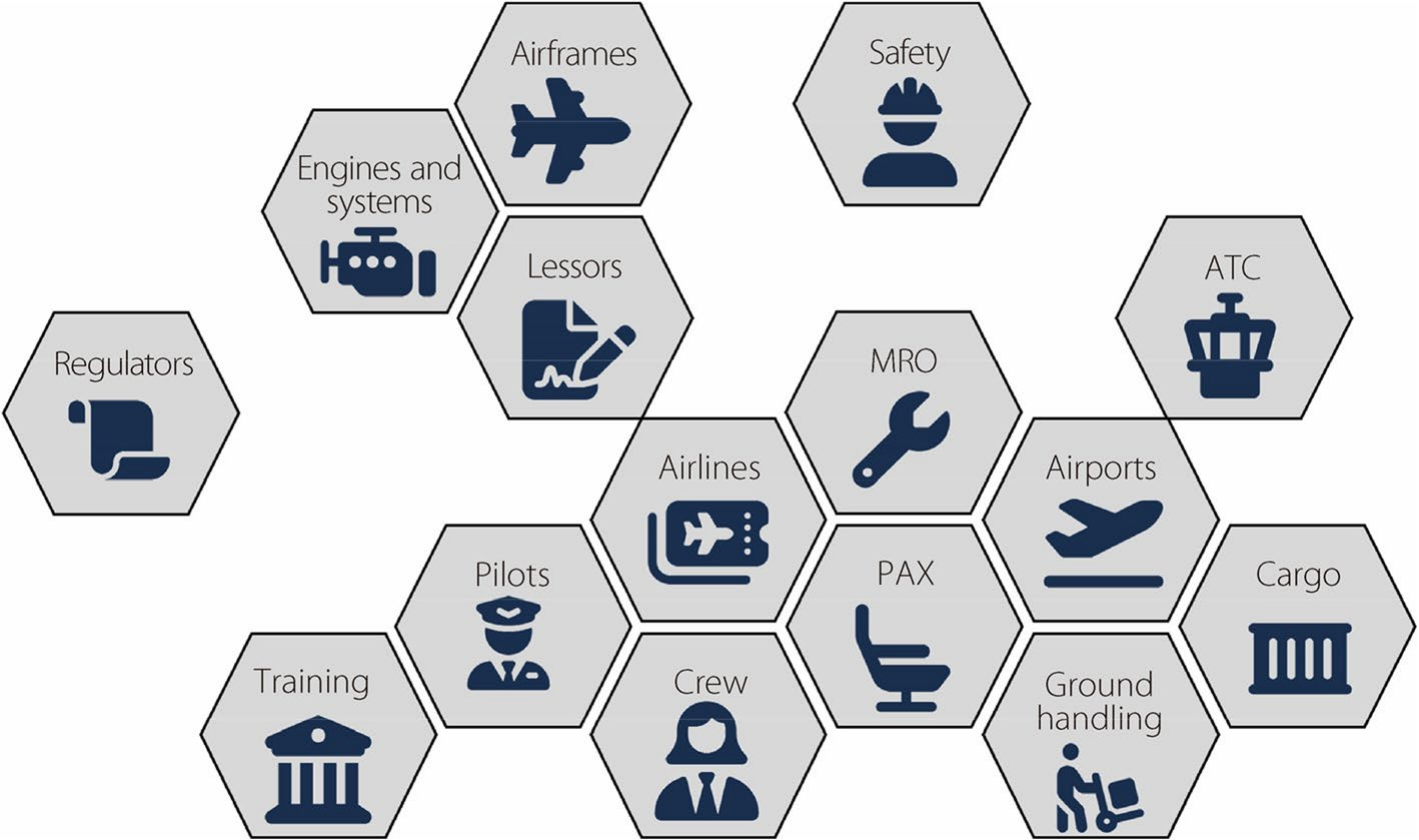
Aspects of the aviation (airline) industry

The aerospace industry encompasses several facets that can be classified into regular passenger (PAX) transport, general aviation, recreational aviation, remotely piloted or autonomous systems, and space [1]. Regular PAX transport, which includes several aspects, is commonly considered in the aviation industry. The results are shown in Fig. 1. Central to all these aspects is safety, which is monitored by regulators such as the Federal Aviation Administration (FAA) and the European Union Aviation Safety Agency. Engineering aspects include airframe designers and manufacturers (e.g., Boeing and Airbus), engine suppliers (e.g., Rolls Royce), and original equipment manufacturers for other systems (e.g., avionics from Honeywell). In aviation, engineering includes maintenance, repair, and overhaul (MRO) services. The most significant owners of aircraft assets are fewer, although some airlines buy and own their own aircraft. On board the aircraft are pilots and cabin crew, in addition to other personnel for the airline in operations, sales, and administration. PAX are clearly carried onboard, and the cabin crew is responsible for numerous aspects of their in-flight experiences. Cargo is another important aspect of the aviation industry. The interface on the ground for everything occurs at the airport, which caters to PAX needs and provides essential services to airlines. Finally, ground and air movements are managed using air traffic control (ATC).

The aviation industry is undergoing a significant transformation driven by rapid advancements in artificial intelligence (AI) and immersive technologies. These innovations have opened new possibilities for enhancing safety, efficiency, and sustainability across various aviation domains, from flight operations and ATC to maintenance and ground handling. Lightweight and mobile AI applications, coupled with technologies such as augmented reality (AR) and virtual reality (VR), are playing a crucial role in this transformation.

AI in aviation is not new; it has been increasingly applied, particularly in forecasting PAX demand and resource allocation. Traditional approaches such as multiple linear regression have been complemented by machine learning (ML) techniques such as artificial neural networks (ANNs), adaptive neuro-fuzzy inference systems, and support vector machines (SVMs) since 2014 [2]. The aviation industry uses AI for various applications, including technology prognosis, market demand prediction, and manufacturing processes [3]. Recent advancements in AI, particularly in areas such as deep learning (DL) and computer vision, combined with the increasing availability of powerful mobile computing devices, have led to a surge in new applications. These applications are not limited to traditional areas but are being extended to aspects such as ground handling, baggage management, and aircraft maintenance. Immersive technologies, such as AR and VR, are also gaining traction, offering new ways to train personnel, guide inspections, and enhance situational awareness. Urban air mobility (UAM) is an emerging area and a key enabling technology [1]. Although AI offers significant potential for improving efficiency and decision-making in aviation, industry professionals are concerned about its impact on future job roles [3].

**Fig. 2:**
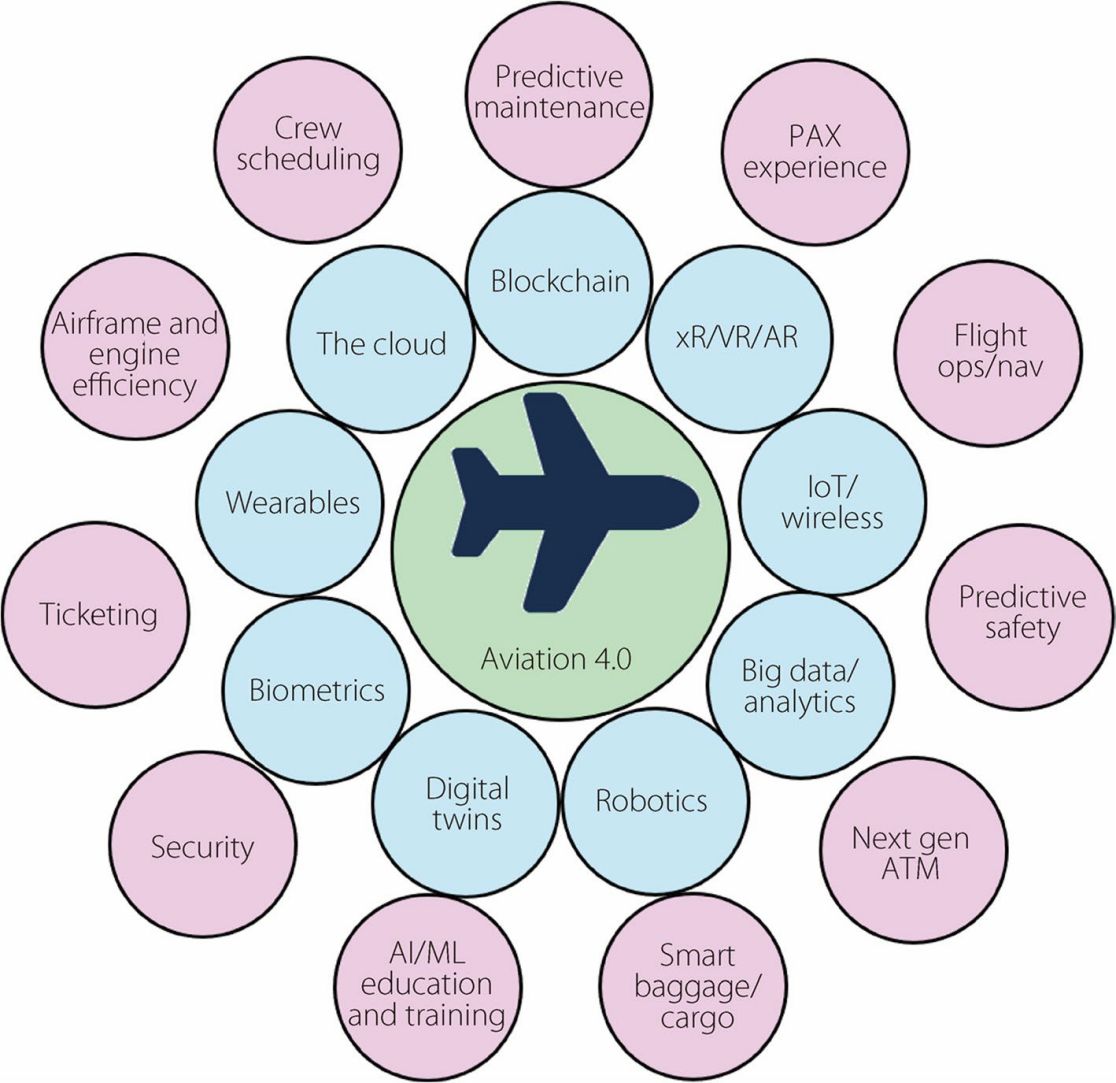
Aviation 4.0 will leverage nine technologies (inner ring) to facilitate eleven industry advancements (outer ring). xR: Extended reality; IoT: Internet of Things

The integration of lightweight mobile AI and immersive technologies has immense significance for the aviation industry [4]. It addresses the pressing challenges related to safety, efficiency, sustainability, and the growing complexity of operations. AI can help mitigate human error, which is a leading cause of aviation incidents [5], through real-time monitoring, pilot assistance systems, and advanced training simulations [6, 7]. AI-driven predictive maintenance can enhance safety and reduce downtime by proactively identifying potential equipment failures [7]. Immersive technologies can provide engaging and realistic training experiences and improve knowledge retention and skill development [8]. Moreover, these technologies contribute to more efficient resource allocation, optimized processes, and reduced environmental impact [9]. Figure 2 highlights these various transformative technologies for the aviation industry, giving Aviation 4.0. Specifically, nine technologies are shown to facilitate 11 significant industry improvements.

To establish the context of this work, a note should be taken from previous review papers that exist in the literature on AI in aviation. These were identified from a Scopus search for “artificial intelligence” and ‘aviation’ in “article title, abstract, keyword,” limited to the document type ‘review,’ with no date range, with the search in March 2025. The reviews are listed in Table 1. Common perspectives across 28 reviews on AI in aviation highlight several key areas. Safety was the predominant theme, with multiple reviews examining the role of AI in enhancing aviation safety and human factors (HFs). Air traffic management (ATM) is another significant area with several studies exploring the potential of AI to improve efficiency and management capabilities. Electric aviation and sustainability are emerging themes that reflect the shift of the industry toward greener technologies.

**Table 1:** Prior reviews on the topic of AI in aviation, giving a general perspective of each

Reference	Year	Title	Perspective
[10]	2025	Applications of natural language processing in aviation safety: a review and qualitative analysis	Safety
[11]	2024	Aircraft surface movement and operation monitoring systems in general aviation and commercial airports: a state-of-the-art review	ATM
[12]	2024	Artificial intelligence in aviation safety: systematic review and biometric analysis	Safety
[13]	2024	A review of the application of fuzzy mathematical algorithm-based approach in autonomous vehicles and drones	Drones
[14]	2024	A review of reinforcement learning for fixed-wing aircraft control tasks	Flight control
[15]	2024	A review of strategies to detect fatigue and sleep problems in aviation: insights from artificial intelligence	Safety/HF
[16]	2024	Airport security: the impact of AI on safety, efficiency, and the passenger experience	Airports
[17]	2024	Digital twins for condition and fleet monitoring of aircraft: toward more-intelligent electrified aviation systems	Electric aviation
[18]	2023	Recent advances in airfoil self-noise passive reduction	Sustainability
[19]	2023	Synergizing machine learning and the aviation sector in lithium-ion battery applications: a review	Electric aviation
[20]	2023	Comprehensive review of battery state estimation strategies using machine learning for battery Management Systems of Aircraft Propulsion Batteries	Electric aviation
[21]	2023	Key technology and future development of regional airliner	Aircraft design
[22]	2022	Review on artificial intelligence techniques for improving representative air traffic management capability	ATM
[23]	2022	Risk management in aviation maintenance: a systematic literature review	MRO
[24]	2022	Research progress and perspective of robotic equipment applied in aviation assembly	Manufacture
[25]	2022	A survey on artificial intelligence (AI) and eXplainable AI in air traffic management: current trends and development with future research trajectory	ATM
[26]	2021	A review on fatigue detection and management for air traffic controllers	ATM/HF
[27]	2021	Research progress and prospects of agricultural aero-bionic technology in China	Drones
[28]	2021	A survey of air combat artificial intelligence	Military
[29]	2021	Overview of aviation big data research	Big data
[30]	2021	Review on basic concept and applications for artificial intelligence in aviation	Military
[31]	2021	Recent trends and challenges in predictive maintenance of aircraft’s engine and hydraulic system	MRO
[32]	2020	Fusion application of DT and AI for aviation intelligent manufacturing	Manufacture
[33]	2018	Research and prospect of intellectualized air traffic management technology	ATM
[34]	2017	Review of control models for human pilot behavior	Safety/HF
[35]	2017	An evolutionary outlook of air traffic flow management techniques	ATM
[36]	2016	Overview of pilot’s associate program	Safety/HF
[37]	2016	Dynamics and control technologies in air traffic management	ATM

This review examines the current status of lightweight mobile AI and immersive technologies in aviation, focusing on their applications, challenges, and future potential. This review aims to answer the following research questions:

- How are AI and immersive technologies currently used in various aviation domains, including flight operations, ATC, maintenance, and ground handling?
- What types of lightweight AI models and hardware considerations are relevant for mobile applications in aviation, particularly in resource-constrained environments, such as drones?
- What are the specific benefits and challenges associated with implementing these technologies?
- What are the future directions and potential of these technologies in transforming the aviation industry?

## Bibliometric analysis

Before moving on to a detailed narrative review, it is worth considering a brief quantitative analysis. Using a bibliometric analysis, we provide insights into the current research efforts related to lightweight and mobile AI and immersive technologies in aviation. By examining various bibliometric indicators, such as keywords, country distribution, and authorship patterns, this quantitative analysis aims to identify trends in the field. This section utilizes network diagrams to visualize these relationships and offers insights into the evolving field of AI and immersive technologies in aviation.

### Methodology

To perform the bibliometric analysis, Scopus was used as the primary database because of its comprehensive coverage of peer-reviewed literature in the fields of science, technology, and engineering. The search was performed using the “Article Title, Abstract, Keyword” search option to ensure a broad and inclusive retrieval of relevant articles. The following search terms were used:

1. (Mobile) AND (Computing) AND (Aviation)
2. (Mobile) AND (AI) AND (Aviation)
3. (Augmented Reality) AND (Aviation)

Note that the brackets indicate a unique search field, for instance, mobile and computer were in separate fields, whereas augmented and reality were in the same field.

The search was performed without a specific date filter to ensure that all relevant literature published up to the present date (November 1st, 2024) was included. The articles were filtered to include only peer-reviewed journal articles, conference papers, and reviews to ensure the quality and reliability of the data. In other words, a PRISMA process was followed, with data screening based on the inclusion of the search terms in the abstract, title, and keywords of the articles.

The collected data were analyzed using VOSviewer, a software tool designed to construct and visualize bibliometric networks. VOSviewer was used to create the network diagrams for each feature of interest.

### Analysis and results

The previously listed search results returned the following number of documents: (1) 184, (2) 18 and (3) 239.

When combined, a total of 426 unique entries were present, with only 15 duplicate entries. A further 23 entries were removed as just proceedings and not explicit publications, resulting in 403 entries used in the final analysis. No further filtering was implemented to process the data; all 403 unique results were included.

#### Keywords

**Fig. 3:**
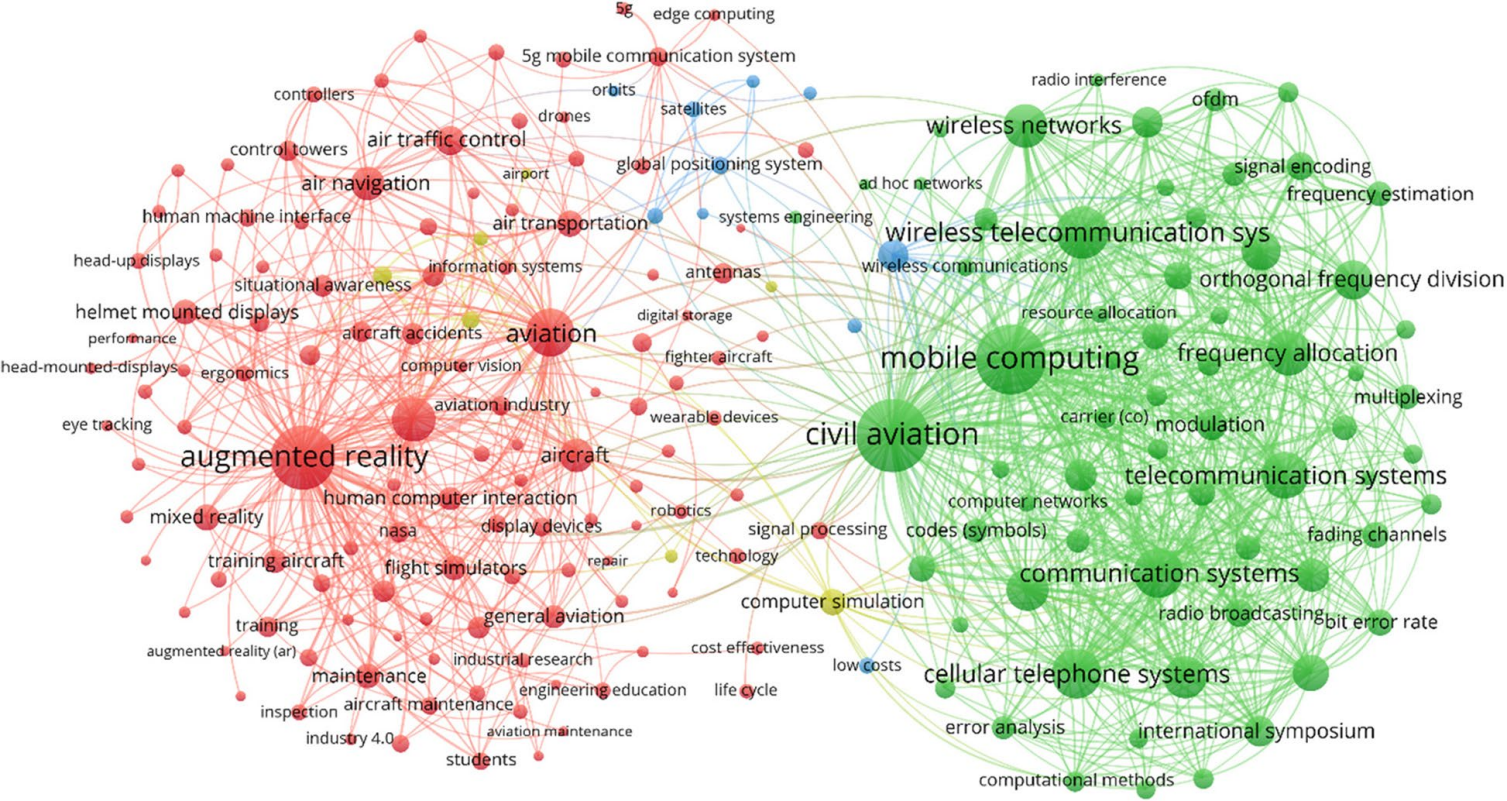
Author keyword co-occurrence

A network diagram of the co-occurrence of author keywords is shown in Fig. 3. Two main clusters are present: the first is around AR (red), which is the largest current application of mobile computing in aviation, and the aviation industry (green) and the associated traditional technology for communication. The red cluster includes several other aspects such as head or helmet mounted displays, human machines or computer interfaces, and direct application areas within the aviation industry (flight training, ATC, and maintenance). From the green cluster, we note the key technologies underpinning the research on mobile computing for aviation, including wireless networks and telecommunication systems. Notable aspects in terms of interference and other issues are also present, which will be active areas of research, given the extreme level of reliability required to utilize technology in aviation. The third cluster captures space-based aspects, specifically navigation instead of communication, which are important for enabling mobile computing globally. A fourth cluster for computing and simulation is also present, but not flight simulation or more simulations of technologies and errors.

**Fig. 4:**
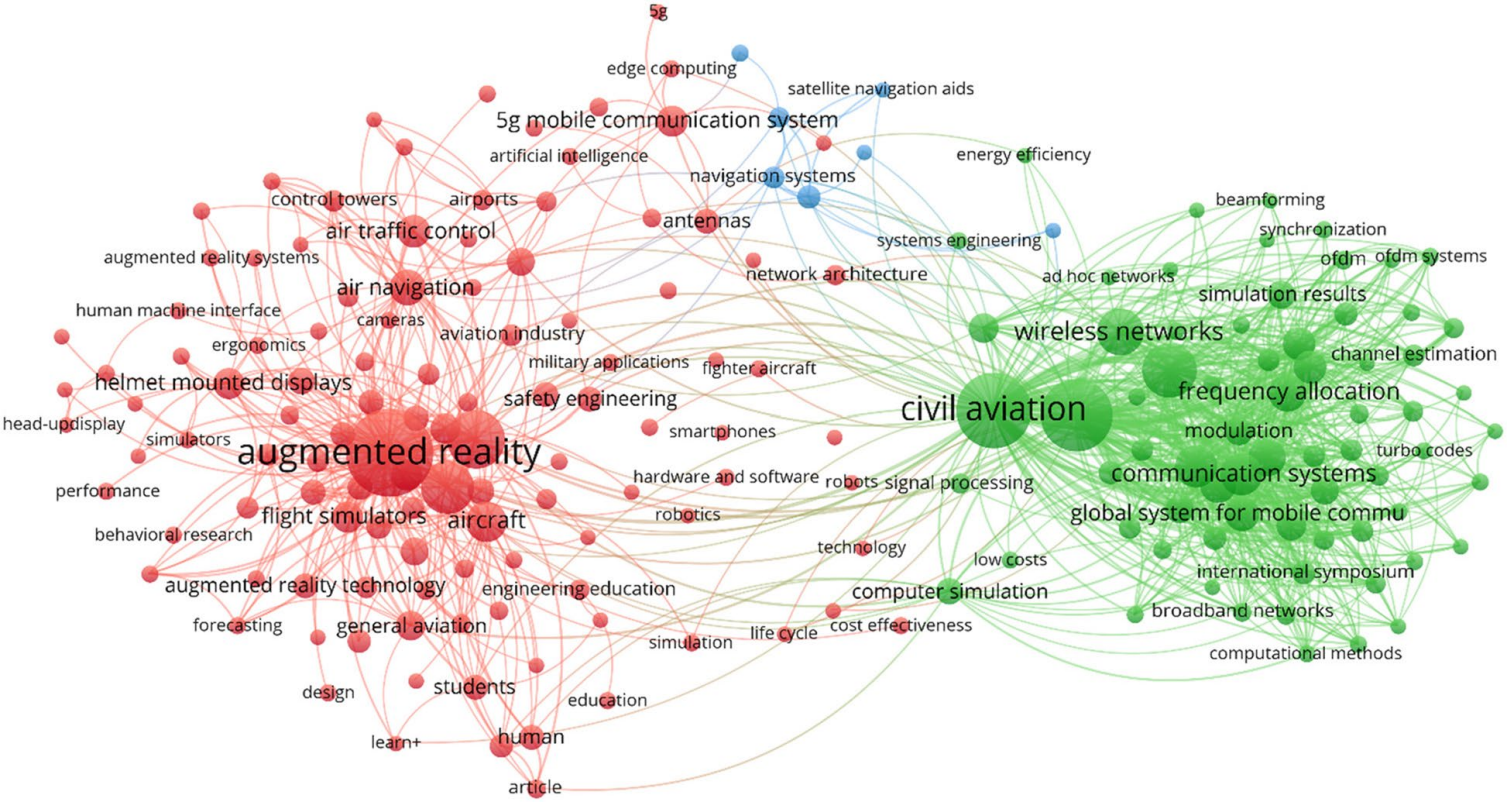
Index keyword co-occurrence

Figure 4 shows the co-occurrence of the index keywords. Analysis of the index keywords revealed results that were similar to those obtained from the author keywords, as shown in Fig. 3. This similarity can be attributed to the manner in which Scopus derives its index keywords. Although author keywords are directly provided by the authors of the articles, Scopus index keywords are assigned based on pertinent information from the article’s title, abstract, and author keywords. The only notable effect is that the fourth smallest cluster (computer simulations) is now incorporated into the green technology cluster, whereas the other three clusters remain the same.

#### Country

**Fig. 5:**
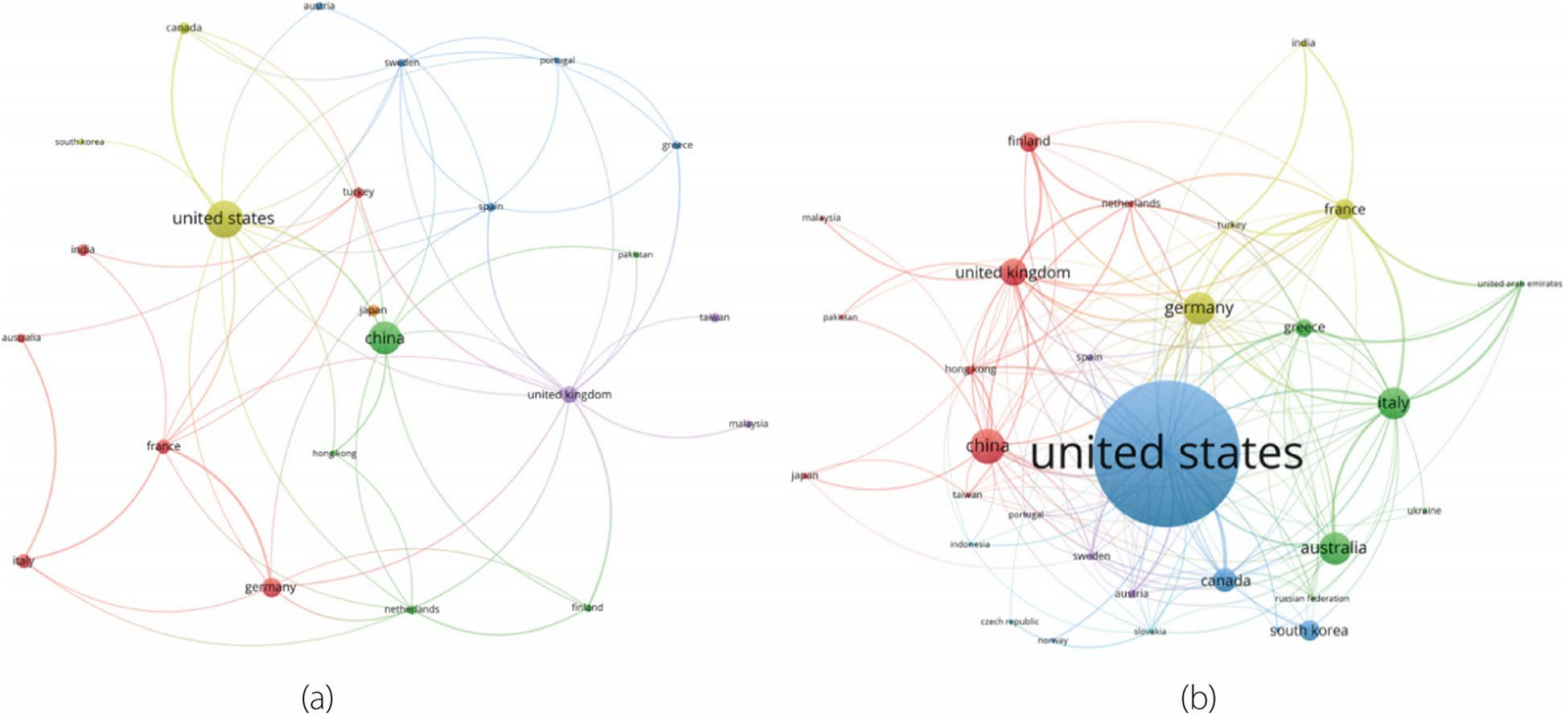
Countries for co-authorship co-occurrence (**a**), and bibliographic coupling by citation (**b**)

Understanding co-authorship and bibliographic coupling by citation for countries offers useful insights into the global research landscape, which are both illustrated in Fig. 5. The co-authorship network (Fig. 5a) highlights direct collaboration and partnerships between countries, showcasing active research efforts. However, bibliographic coupling by citations (Fig. 5b) revealed thematic connections and shared research interests, indicating how countries influence and are influenced by similar research themes. Together, these measures provide a well-rounded view of direct and indirect research relationships that can foster international collaboration and advance scientific knowledge.

In the co-authorship network, the United States and China are similar in size, indicating that they share a similar proportion of research that has been conducted. This was followed by Germany and the United Kingdom in addition to several other countries responsible for smaller proportions of research. In contrast, for bibliographic coupling by citation, the larger size of the United States node clearly suggests that its research is cited more frequently in the literature, followed by China. Interestingly, some smaller countries are now much larger, with Australia exhibiting the most significant increase. This suggests that, although Australia does not conduct as much research, it is of high quality, such that it is well cited.

## AI in aviation education and training

**Fig. 6:**
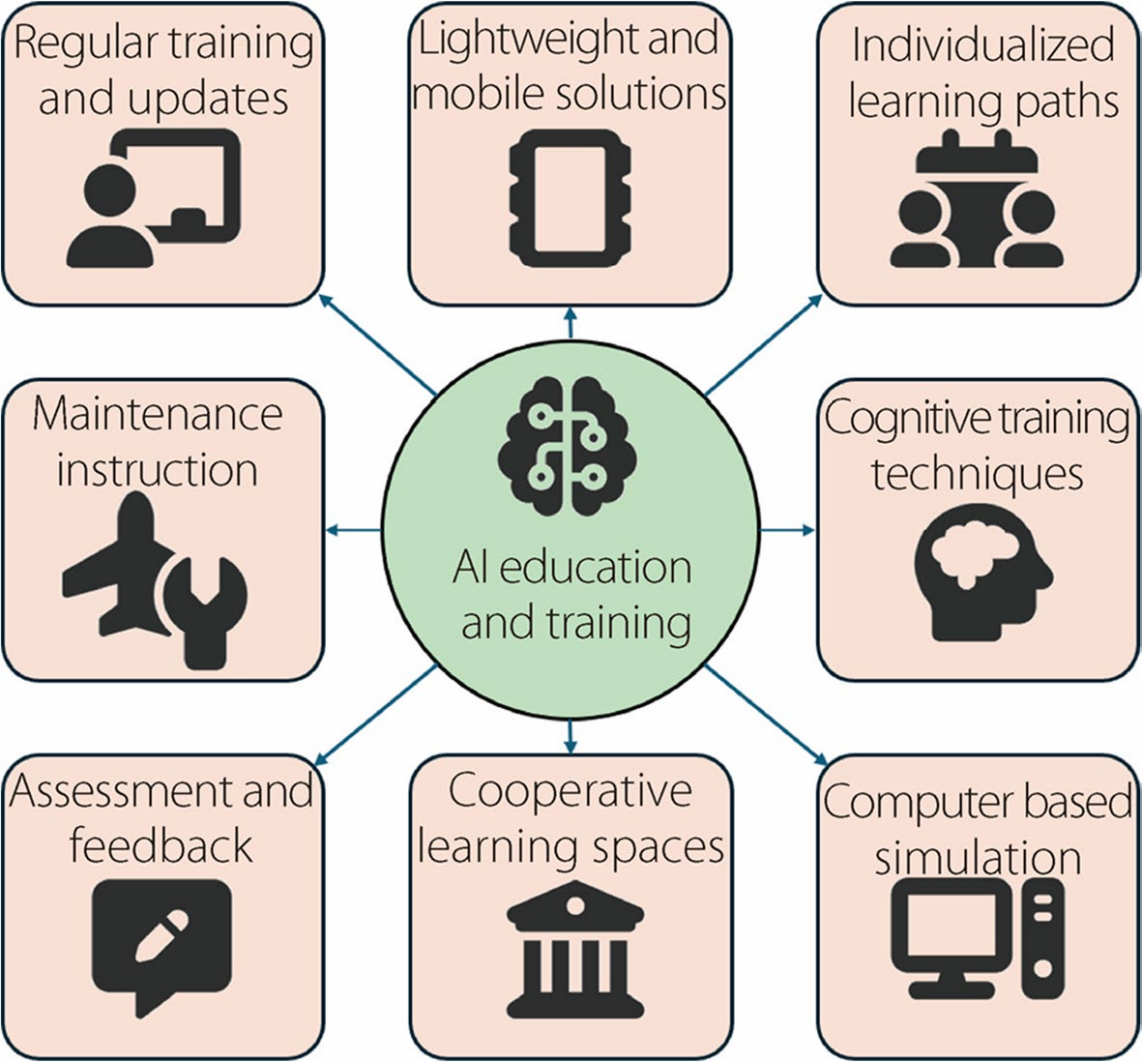
AI applications in aviation education and training

AI is being integrated into an increasing number of aviation education sectors to enhance the entire learning process and make it more efficient, adaptable, and sensitive to the shifting needs of both students and the aviation industry [38]. Moreover, by offering customized and adaptable learning opportunities, this technology revolutionizes training and pilot assessment strategies. Traditional assessment procedures are enhanced by automated, real-time feedback, which ensures that pilots receive personalized instructions that enable them to advance their abilities more rapidly and precisely. Figure 6 shows the implications of AI in aviation education and training process.

### General education

A recent review of aviation education programs in higher education aimed to identify the basic AI capabilities required across the aviation field and improve curricula [4]. The study found that the current bachelor’s level curricula give little weight to developing conceptual understanding and practical expertise in basic AI technologies. By recognizing students’ insights and conveying their concerns, policy makers can establish erudite guidelines and schemes to implement AI tools effectively to enhance teaching and learning in higher education [39]. The impact of AI on aviation education in terms of the utilization of ChatGPT and AI-powered assistants was investigated, and improvements were observed in student learning, writing skills, and time management using these technologies [40]. The progression of aviation education from conventional to technology-driven methods emphasizes the importance of maintaining a delicate balance between human competence and technical advancements [41].

AI applications in education can assist in addressing modern problems using technologies such as intelligent tutoring systems, smart learning, and social robots [42]. An overview of AI in education has been provided, which integrates learning sciences with AI to allow adaptive learning environments and improve education through digital technology [43].

### Uncrewed systems for AI and mobile computing in aero education

The inclusion of drone technology can significantly improve the theoretical information of students and encourage them to have hands-on experiences. Moreover, the practical implications have a greater impact on the understanding of AI [44]. The education system must be altered to enhance the efficacy of training programs for highly qualified staff in the production and operation sectors of uncrewed aerial systems (UASs) by introducing advanced technologies and learning methods [45]. To improve their professional careers in the field of UASs, students’ performance in research projects that focus on AI and control of UASs, as well as crash identification and evasion systems for UASs, should be assessed [46].

Moreover, project-based learning methods have been developed for airport system designs to benefit engineering education by exploring new inventions [47]. Students seeking careers in aeronautical engineering were given access to a flexible project-based program and hands-on platform [48]. This demonstrated that a thorough experiment based on UASs visual navigation enhanced the students’ professional expertise and stimulated their interest in independent exploration.

A novel approach for evaluating the efficacy of drone operation training was also suggested [49]. VR and outdoor experiments on drone flying were used to collect physiological data. Furthermore, the performance of drone operators employing ML models, mental workload, and stress was estimated. Other studies examined techniques based on ML models to estimate the mental workload of pilots throughout a training session [50].

### Aviation safety training

Both AI and VR can be integrated into aviation safety training in aviation [51]. This study analyzed the aspects of hazardous events in aviation, created a scenario for risky incident drills using VR technology, and visualized the emergency response to such events. The application of neural networks to evaluate the accuracy and timeliness of air traffic controllers in decision-making has also been presented [52]. This study introduced AI-integrated training environment that helps operators develop decision-making skills in situations of stress and uncertainty.

### Flight simulator training

A system was proposed to collect data from flight simulators and evaluate pilot performance using AI algorithms [53]. Signal processing combined with ML provides a framework for analyzing flight data and deriving insights into pilot maneuvering [54]. A dynamic Bayesian network-based model enhances pilot assessment by analyzing flight phases [55]. Supervised learning techniques enable trainers to monitor student performance and identify their strengths and weaknesses [56]. Another model combines fuzzy set theory and knowledge analysis to improve aviation education [57].

Cognitive features help to identify high-risk pilots [58]. Intelligent software agents train and evaluate pilots’ situational perception by observing physiological cues such as eye movements [59]. Supervised learning methods examine pilots’ sensory organs for safe flights, and a model identifies student pilots’ vestibular vision with improved accuracy [60]. The association between pilot behavior and brain activity was studied using supervised learning by measuring physiological signal variations during turning tasks [61]. ML classifies pilot training environments based on physiological responses [62].

The new technique identifies alerts in flight simulators, enhancing safety by identifying attention lapses in real time and providing feedback [63]. One training program demonstrated efficacy changes with AI and VR technologies [64]. An AI-based tutoring system that uses a virtual teacher in a flight simulator provides better outcomes [65]. The instructor offered visual feedback, and the virtual instructor system provided customized feedback [66]. Speech recognition in flight simulators using neural networks is another research topic [67].

Automation and AI in flight crew training can reduce the time and expense required to create training materials and improve skill standards [68]. Reinforcement learning (RL) and autoencoders (AEs) pinpoint failure locations and supply command inputs to aircraft control systems, thereby enabling appropriate maneuvers to counteract failures [69]. A simulation-based pilot training framework using RL shows AI advancements offer excellent training prospects [70].

AI-powered simulations, data analytics, and ML algorithms have been used to develop customized training programs that allow trainees to experience real flight cases and receive tailored feedback [71]. A thorough analysis of big data and AI applications in pilot training was completed [72]. Formal language and an electronic flight instructor can automate flight training [73]. An off-axis flight visual display system using deep neural networks (DNNs) focuses on pilot training from takeoff to landing [74]. ML detects students at greater attrition risk during various training phases [75]. AI systems identify maneuvers, evaluate them, and classify trajectory data [76].

An intelligence-based tutoring program has been suggested [77] that helps students become proficient in entrance maneuvers, with AI tutors providing one-on-one guidance [78]. AI integrated with competency-based training and assessment implements intelligent human system integration for airline personnel [79]. Similarly, a virtual simulation-pilot engine using AI accelerates air traffic controller training by recognizing and reacting to verbal communication [80]. ML and VR distinguish expert pilots from beginners, thus improving pilot selection strategies [81].

### Summary and case study

Table 2 provides an overview of the key findings of recent studies on the integration of AI into aviation education. It highlights the main areas of focus, including general education, uncrewed systems, aviation safety training, and flight simulation training. The table outlines the key points, technologies and methods used, and the impact or findings for each section, offering a summary of the use of AI to enhance various aspects of aviation education.

**Table 2:** Summary of AI for aviation education

Section	Key point	Technology/method	Impact/finding
General education	Review of aviation education programs Need for AI capabilities in curricula Impact of AI tools like ChatGPT	AI-powered assistants Intelligent tutoring systems Smart learning Social robots	Improved student learning, writing skills, and time management Balance between human competence and technical advancements
Uncrewed systems for AI and mobile computing in Aero Eng education	Inclusion of drone technology Need for advanced training programs Project-based learning methods	Drone technology VR ML models	Enhanced theoretical and practical understanding Improved professional expertise and independent exploration Assessment of mental workload and stress
Aviation safety training	Integration of AI and VR in safety training Analysis of hazardous events AI integrated training environment	VR Neural networks	Improved decision-making skills in stressful situations Enhanced emergency response visualization
Flight simulator training	Data collection and evaluation using AI Cognitive feature analysis Intelligent tutoring systems	Signal processing ML Dynamic Bayesian networks Fuzzy set theory Supervised learning Speech recognition RL AEs	Improved pilot performance and assessment Identification of high-risk pilots Customized training programs Enhanced safety and feedback mechanisms

To illustrate the practical application of AI in aviation education, we conducted a case study focusing on the use of an AI flight instructor [38]. This study demonstrated that an AI flight instructor significantly improved the simulator performance of ab initio student pilots. The AI instructor provided personalized guidance and support by leveraging advanced AI algorithms and real-time feedback mechanisms.

## AI for aviation safety

**Fig. 7:**
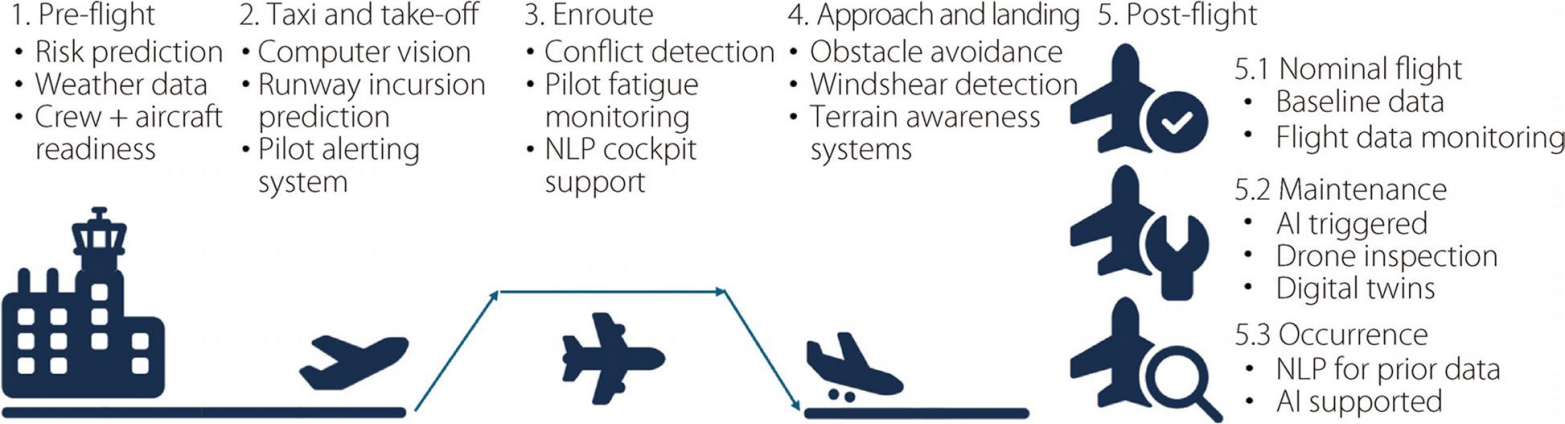
AI-supported safety functions during each flight phase

The aviation industry is an intricate field that combines rigorous operational demands with uncompromised safety standards. Maintaining safety has become a pressing challenge with the increasing air traffic and complexity. Human errors, unpredictable weather, mechanical issues, and coordination breakdowns contribute to these incidents, highlighting the need for proactive and reliable systems [82]. AI has emerged as an important tool for addressing these challenges using data analytics, ML, and automation to enhance safety [83]. Figure 7 illustrates how AI might support aviation safety across all phases of flight, from pre-departure risk prediction to post-flight analysis, encompassing both routine operations and safety-critical events.

### Importance of AI in aviation safety

The unique ability of AI to process large volumes of real-time data is vital for safety-focused applications in aviation. Technology not only aids in detecting potential failures and safety threats but also provides predictive insights and decision-support systems that enhance the reliability of operations. By analyzing past incidents, monitoring equipment in real time, and aiding human operators in complex decision-making, AI optimizes safety protocols to help prevent incidents before they occur [84]. In a sector where a single mishap can have tragic consequences, AI-driven systems are invaluable for preemptive actions and for supporting pilots, engineers, and air traffic controllers.

### Pilot assistance and autonomous systems: AI as a copilot

The integration of AI into cockpit systems has introduced enhanced pilot assistance tools that significantly improve safety. In challenging situations, such as severe weather or technical failures, AI systems analyze flight data and provide real-time insights and recommendations, assisting pilots with difficult decision-making and reducing their cognitive load. The use of AI in pilot assistance has proven particularly valuable in scenarios where quick and precise responses are essential to prevent accidents [85].

Autonomous systems are gradually advancing with projects such as Airbus’s Autonomous Taxi, Takeoff, and Landing (ATTOL) project, which aims to bring automation to critical flight stages. ATTOL showcases the potential of autonomous flight systems using AI for navigation and decision-making, thus reducing the risk of human error [86, 87]. Although fully autonomous flights remain a goal, AI-powered systems with limited automation capacities already support pilots in high-stress environments.

However, the shift toward autonomy is accompanied by challenges, particularly in managing the relationship between human pilots and AI. Ensuring that pilots remain engaged while AI handles certain tasks is crucial, because disengagement can lead to skill degradation over time. Therefore, AI-driven tools prioritize collaboration, where pilots retain authority while AI acts as a supportive copilot, thereby enhancing safety without compromising human oversight [88].

### Mitigating human error through AI: reducing risks in complex environments

Human errors remain a leading cause of aviation incidents. AI can address this issue by identifying common risk factors and providing actionable insights into risk mitigation. For instance, AI-driven simulation training programs that incorporate xR technology allow pilots to rehearse rare but critical scenarios in controlled environments. These simulations include unexpected events such as engine failure, severe turbulence, and emergency landings, providing pilots with practice in handling complex situations and enhancing their response times and decision-making skills [89].

Beyond training, AI aids in real-time error prevention through systems that monitor pilot actions and provide immediate feedback. For instance, in cases where a pilot might miss a critical checklist item, AI can detect an oversight and prompt corrective action, thereby minimizing the risk in real time. This capability is particularly effective in multi-tasking environments, where pilots may experience cognitive overload, as it reinforces critical procedural adherence without distracting them from essential flight tasks.

### AI in safety management systems: streamlining risk management

Safety management systems (SMSs) are essential for safety efforts in aviation organizations. AI complements SMS by streamlining risk assessments, identifying safety trends, and enabling predictive safety analyses. AI algorithms can analyze historical data to identify correlations between operational practices and safety incidents, helping organizations prioritize safety initiatives based on real-time and predictive risk assessments [90].

Predictive risk models powered by AI provide insights into potential hazards, enabling airlines to allocate resources to at-risk areas. For instance, if data from past incidents suggest an increased likelihood of accidents under certain weather conditions, airlines can plan contingencies to improve response times. By fostering data-driven decision-making, AI augments the effectiveness of SMS frameworks, ensuring a proactive approach to safety management.

### Accident prevention and incident analysis: the role of AI in post-incident investigations

AI is instrumental in accident prevention and plays a critical role in post-incident analysis, where rapid and accurate insights are vital. Following an incident, AI tools can process large volumes of data, including flight data records, maintenance logs, and weather reports, to identify root causes. Through ML and natural language processing (NLP), AI systems extract relevant details from unstructured data sources, quickly identifying contributing factors and recommending preventive measures [91, 92].

AI-driven incident analysis helps aviation authorities and organizations learn from each event and continuously refine safety protocols to prevent recurrence. For instance, NLP models can analyze historical incident reports and reveal commonalities in causal factors, offering valuable insights into shaping future training, operational protocols, and safety policies. This capability complements traditional investigation methods and enhances the ability of the industry to adapt and evolve in response to safety challenges.

### Ethical considerations and challenges: addressing the role of AI in critical decisions

Despite its benefits, the integration of AI into aviation safety introduces ethical and regulatory challenges. The role of AI in safety-critical decisions where lives are at stake, raises questions about accountability and responsibility [93]. In situations in which AI may contribute to a decision with negative consequences, determining the fault becomes complex. Regulatory bodies, including the International Civil Aviation Organization, are developing guidelines to ensure the reliability, transparency, and accountability of AI.

Algorithmic bias is another concern. AI systems rely on historical data and any bias in these data could inadvertently lead to skewed outcomes, potentially affecting safety [88]. Furthermore, the cybersecurity risks associated with AI systems require robust protection to prevent malicious attacks that could compromise safety. Establishing a strong data governance framework, along with stringent regulatory oversight is essential to ensure that AI systems operate with fairness, transparency, and security.

### Summary and case study

Table 3 summarizes an overview of the key findings of recent studies on the role of AI in enhancing aviation safety. It highlights the main areas of focus, including the pilot assistance and autonomous systems, mitigating human error, AI in SMS, accident prevention and incident analysis, and ethical considerations and challenges. The table outlines the key points, technologies and methods used, and the impact or findings for each section, offering a summary of how the utilization of AI to address safety challenges in the aviation industry.

**Table 3:** Summary of AI for aviation safety

Section	Key point	Technology/method	Impact/finding
Pilot assistance and autonomous systems: AI as a copilot	Enhanced pilot assistance tools Real-time insights and recommendations Autonomous systems like Airbus’s ATTOL project	AI-powered pilot assistance Autonomous navigation and decision-making	Improved safety in challenging situations Reduced risk of human error Collaboration between pilots and AI
Mitigating human error through AI	Identifying common risk factors AI-driven simulation training Real-time error prevention	xR technology Real-time monitoring and feedback	Enhanced response times and decision-making Minimized risk through immediate corrective actions
AI in SMSs	Streamlining risk assessment Identifying safety trends Enabling predictive safety analyses	AI algorithms Predictive risk models	Data-driven decision-making Proactive safety management
Accident prevention and incident analysis	Post-incident analysis Identifying root causes Recommending preventive measures	ML NLP	Rapid, accurate insights Continuous refinement of safety protocols
Ethical considerations and challenges	Accountability and responsibility in safety-critical decisions Algorithmic bias Cybersecurity risks	Regulatory guidelines Data governance frameworks	Ensuring AI reliability, transparency, and security

Several case studies have demonstrated the successful application of NLP in enhancing aviation safety practices [10]. For instance, NLP algorithms have been used to analyze incident reports from the Aviation Safety Reporting System, extracting key insights from narrative data and identifying latent safety risks and contributing factors [94]. Topic modeling has also been used to analyze aviation incident narratives from the Australian Transportation Safety Bureau dataset by employing techniques such as probabilistic latent semantic analysis, latent semantic analysis, latent Dirichlet allocation, and non-negative matrix factorization to extract valuable insights and highlight their distinct advantages and limitations in aviation safety analyses [91]. Further studies showed that NLP and AI models, such as residual networks and stochastic recurrent neural networks, can classify flight phases from NTSB safety reports with over 68% accuracy, thereby outperforming random guesses [95]. A related study highlighted the role of dataset size in predictive model performance, with larger datasets consistently yielding higher accuracy, emphasizing the need for improved data collection processes in aviation safety [96].

## ATC and ground handling

**Fig. 8:**
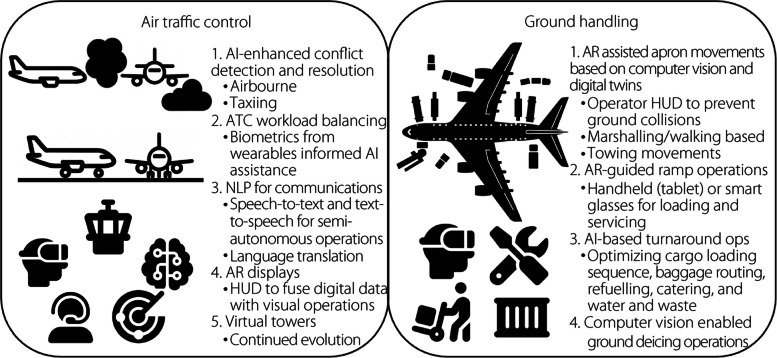
AI and mobile/immersive technologies for ATC and ground handling

The use of AI and ML systems is likely to significantly alter airport ground handling, airside operations, and ATM. Presently, all these operations, particularly airside oversight, are highly reliant on human operators. The use of smart machines and other forms of enhanced computer-based automation, alone or in combination with human operators, offers a means of increasing the efficacy and efficiency of such operations. Several of such systems, such as those in other industrial applications, will have to work autonomously without direct oversight from the human crew, while seamlessly coordinating with airside systems that are still under human control (i.e., trusted autonomy). The potential exists for discrete AI solutions as well as the integration of applications from other areas and industries. Opportunities exist to integrate autonomous vehicles, autonomous monitoring, coordination of required resources, and efficient allocation of scarce resources. In particular, given the increasingly complex and congested ground and airspace in the vicinity of major airports, allocating ramp space, logistical support, and airspace (e.g., efficient flight path routing) are all potential applications. For instance, AI is being explored for aircraft docking, potentially impacting ground marshalling jobs and necessitating personnel to adapt and develop AI-related skills [97]. Figure 8 illustrates how AI and lightweight mobile computing technologies, including AR and VR, will increasingly be used to support safety-critical and efficiency-focused tasks in both ATC and ground handling.

### Baggage handling

Beginning with the airside AI-powered solutions closest to the landside, the tracking, identification, monitoring, and securing of baggage can benefit significantly from the integration of machine vision and AI [98]. Specifically, machine vision and blockchain technology have been suggested as a means of accurately identifying individual bags and tracking them throughout their journey from the check-in counter to the aircraft, significantly reducing the risk of lost or misrouted luggage [99]. During this process, baggage can be continuously monitored to ensure that any discrepancies are detected and addressed [98, 100]. Although addressing such discrepancies is currently considered a role of humans, it may eventually be performed by smart machines, similarly to other roles. For PAX, AI offers both direct benefits and convenience. For instance, research has shown that an AI-powered tracking system that uses radio frequency identification (RFID) technology can increase baggage reclamation efficiency by over 70% [101].

Beyond ensuring the physical security of baggage, machine vision in baggage handling systems allows the verification of the items themselves. In the aviation context, both nefarious and non-nefarious baggage threats exist [102, 103]. Threat detection systems based on machine vision and other AI solutions are primarily designed to detect weapons [104]. Weapons, such as handguns, can be detected using edge-based features and SVMs [105]. However, higher precision is possible using more advanced techniques that analyze multi-view X-ray scans, enabling the accurate detection of guns, blades, and bottles [106–109]. Using of three-dimensional (3D)-X-ray imaging and advanced descriptors (e.g., SIFT [110] and HOG features [111]), anomalies such as overheating or irregular shapes can be detected. Such systems are capable of detecting both nefarious (e.g., guns) and non-nefarious (e.g., lithium batteries in thermal runaway) threats to aviation safety. Detection rates can be further enhanced using RL approaches or active vision frameworks [112, 113]. These approaches enable continuous real-time monitoring that is beyond the capabilities of human operators.

The transport of travelers’ baggage and other cargo carried by aircraft from terminal buildings and outbuildings to aircraft, baggage trucks, and other transport vehicles is required [114, 115]. In such complex, dynamic, and busy environments, the use of AI and ML models for optimal routing, dynamic allocation, and load balancing presents significant opportunities for enhancing efficiency and effectiveness [116]. Human operators may face substantial delays in performing seemingly straightforward tasks, such as driving from one location to another in an airfield, when these vehicles must cross paths with other ground vehicles and maneuvering aircraft. Understanding the dynamics of every vehicle operating in a major aerodrome is essentially impossible for human operators or even large teams. AI algorithms, which can analyze location, movement, and future positioning based on scheduling data, can identify routes that are not apparent to human operators [115]. These systems minimize delays and fuel consumption [116].

The increasing use of automatic baggage systems has not fully displaced the need for physical handling of baggage within the system, particularly during the ‘manhandling’ of baggage during loading and unloading [117]. Given the continued reliance on physical labor within airside operations, particularly at smaller airports lacking automated baggage systems, the health and safety of baggage handlers are critical concerns. Fortunately, AI, combined with wearable devices and sensors, presents new opportunities for monitoring and improving worker health [118]. Although such applications are yet to be applied within space, the literature already shows how a combination of sensors and AI can monitor ergonomics during physical exertion and enable reductions in musculoskeletal strain [119]. These approaches can be used to predict injury risk [120] and provide real-time feedback to workers to alter their behavior [121].

### Airside ground movements and safety

Considering the need to replace fossil fuel-powered vehicles with cleaner electric models [122], optimal routing is essential to manage the different energy flexibilities of such vehicles [123]. Although not a primary focus in the aviation literature on airside operations, the dynamic allocation of electric vehicles will also become imperative. Fortunately, literature from other fields offers insights into the dynamic allocation of electric vehicles, particularly given the charging times [124] and the need to manage the demands placed on local electrical grids [125]. Using ML models, one can manage the fleetwide state of charge [126] and ensure load balancing across the fleet [127].

The routing of vehicles airside is not limited to supporting vehicles, but includes taxi routing of aircraft. The use of multi-agent systems that can adaptively reroute aircraft based on real-time temporal and spatial data from multiple data sources can prevent delays and reduce fuel consumption [128]. Such systems are more suited to this than humans, given the vast complexity of traffic flow data and the dynamics of airside environments. The use of AI-enabled dynamic taxi route adjustments can account for taxiway utilization, runway availability, and conflicts between aircraft and support vehicles [129]. Further optimization is possible using algorithms such as mixed-integer linear programming (MILP), enabling the balancing of runway and taxiway allocation, thereby minimizing bottlenecks through predictive and preventative awareness [130]. Even the assignment of airport gates can be improved with the smart data approach, resulting in the minimization of gate blockage time [131].

To ensure that the aircraft can safely taxi and takeoff, the aerodrome surfaces must remain clear of foreign object debris (FOD). Traditionally, FOD detection has relied on human visual inspection, although technological advances have introduced more automated methods [132]. Computer vision algorithms, such as YOLO [133], combined with drones, can detect and track FOD in real time with greater speed and accuracy than human operators [134, 135]. It is also important to consider that, in the future, through trusted autonomy, such drone-based systems could operate independently from humans while coordinating with flying systems that are still controlled by human operators. AI-driven models that use computer vision are also capable of automatically detecting new risks based on real-time data, such that airside safety tasks can be completed more autonomously and at lower costs than at present [135].

### ATM

In ATM, opportunities exist for the integration of AI and ML. Greater efficiency and safety in ATM can be achieved using AI-driven models to analyze and optimize aircraft trajectories, along with arrival and departure sequencing. Using real-time spatial and temporal data, such AI-based systems can predict and analyze highly dynamic systems and provide more efficient routing and allocation, particularly in high-traffic environments around major airports [22, 136]. Runway sequencing and congestion, and therefore delays, can be reduced through the application of advanced algorithms such as branch-and-bound [137]. Traffic flow management can also be enhanced through ML models (e.g., random forest) to find sources of inefficiency and optimize route planning [136]. In combination, such AI solutions can significantly streamline operations at and around major airports, enabling more precise decision-making [138], with clear safety and commercial benefits. Reductions in taxi and flight time, including holding time, can also reduce the impact of such operations on the natural environment.

As the volume of global air traffic increases, managing airspace efficiently becomes increasingly complex. AI is being integrated into ATM systems to optimize routes, minimize delays, and reduce safety risks associated with congestion. Using advanced algorithms, AI-based systems process real-time data on air traffic flow, weather, and aircraft positioning to make rapid adjustments, thereby reducing the risk of mid-air collisions and ensuring smooth operation [139].

A key advancement in this field is the use of AI for trajectory prediction. AI assists in coordinating movements across congested airspaces by anticipating the flight paths of multiple aircraft [93]. For instance, the FAA NextGen program leverages AI to enhance route efficiency and reduce airborne holding times, thus reducing both fuel consumption and risk factors linked to air congestion [140].

The Single European Sky ATM Research (SESAR) project in Europe is another example of the application of AI to ATM. SESAR has successfully used AI to facilitate safer and more efficient ATC, thereby reducing the risk of miscommunication and enhancing the situational awareness of air traffic controllers. By integrating data from various sources, including radar and flight plans, SESAR’s AI-powered systems improve controller response times to unforeseen events, ultimately supporting safer skies [139].

Several recent reviews have focused on the use of AI in ATM. A systematic literature review, supplemented by bibliometric analysis, reveals the growing application of AI in air transport, particularly in prediction, optimization, and inter-industry collaborations, while highlighting the need for further research on ethics, legality, integration, and environmental impact [141]. Another review examined the application of AI techniques to ATM, highlighting their advantages in computational efficiency and alternative problem-solving approaches while identifying future research directions for model selection and architecture optimization for specific ATM missions [22]. The next review explored the current applications of AI and explainable artificial intelligence (XAI) in ATM; identified key trends and challenges; and outlined future research directions for enhancing the safety, efficiency, and transparency of air transportation [25]. A final review focused on the application of AI and ML techniques in ATC, particularly in conflict detection and resolution, emphasizing the potential of AI to enhance automation and efficiency in ATM operations [142].

### Summary and case study

Table 4 provides an overview of the key findings of recent studies on the integration of AI into ground handling and ATC operations. It highlights the mainly focus areas including baggage handling, airside ground movements, safety, and ATM. The table outlines the key points, technologies and methods used, and the impact or findings for each section, offering a summary of the utilization of AI to enhance efficiency, safety, and operational effectiveness in these critical areas of aviation.

**Table 4:** Summary of AI for ATC and ground handling

Section	Key point	Technology/method	Impact/finding
Baggage handling	Tracking, identification, monitoring, and securing of baggage Detection of nefarious and non-nefarious threats Optimal routing and load balancing Health and safety of baggage handlers	Machine vision Blockchain technology RFID technology Multi-view X-ray scanning RL Wearable devices and sensors	Reduced risk of lost or misrouted luggage Increased baggage reclaim efficiency Enhanced threat detection Minimized delays and reduced fuel consumption Improved worker health and safety
Airside ground movements and safety	Replacement of fossil-fuel-powered vehicles with electric models Dynamic allocation of electric vehicles Taxi routing of aircraft FOD detection	ML models Multi-agent systems MILP Computer vision algorithms Drones	Optimized routing and load balancing Reduced delays and fuel consumption Improved gate assignment Enhanced FOD detection and airside safety
ATM	Optimization of aircraft trajectories and sequencing Real-time data analysis for efficient routing Trajectory prediction Enhanced situational awareness for air traffic controllers	AI-driven models Advanced algorithms (e.g., branch-and-bound) ML models (e.g., random forest) Trajectory prediction AI-powered systems (e.g., FAA’s NextGen, SESAR)	Reduced delays and congestion Improved route efficiency Enhanced safety and situational awareness Reduced environmental impact

As a case study, SATS, the primary ground-handling service provider at Singapore Changi Airport, introduced AR smart glasses to enhance ramp handling operations [143]. This provides ramp handling staff with critical information, such as loading instructions, in real time. Using wearable AR technology, operators can scan visual markers on baggage and cargo containers to obtain details such as weight, unit number, loading sequence, and allocated position within the aircraft. The implementation of AR smart glasses has significantly improved the accuracy and efficiency of baggage and cargo loading. This hands-free process enhances safety and allows for better supervision and instruction from flight controllers within the control center. This technology has also increased productivity, potentially shortening loading times by up to 15 min, which benefits airline customers by reducing waiting times for PAX and shortening transit times for airfreight shippers.

## Design and maintenance engineering

**Fig. 9:**
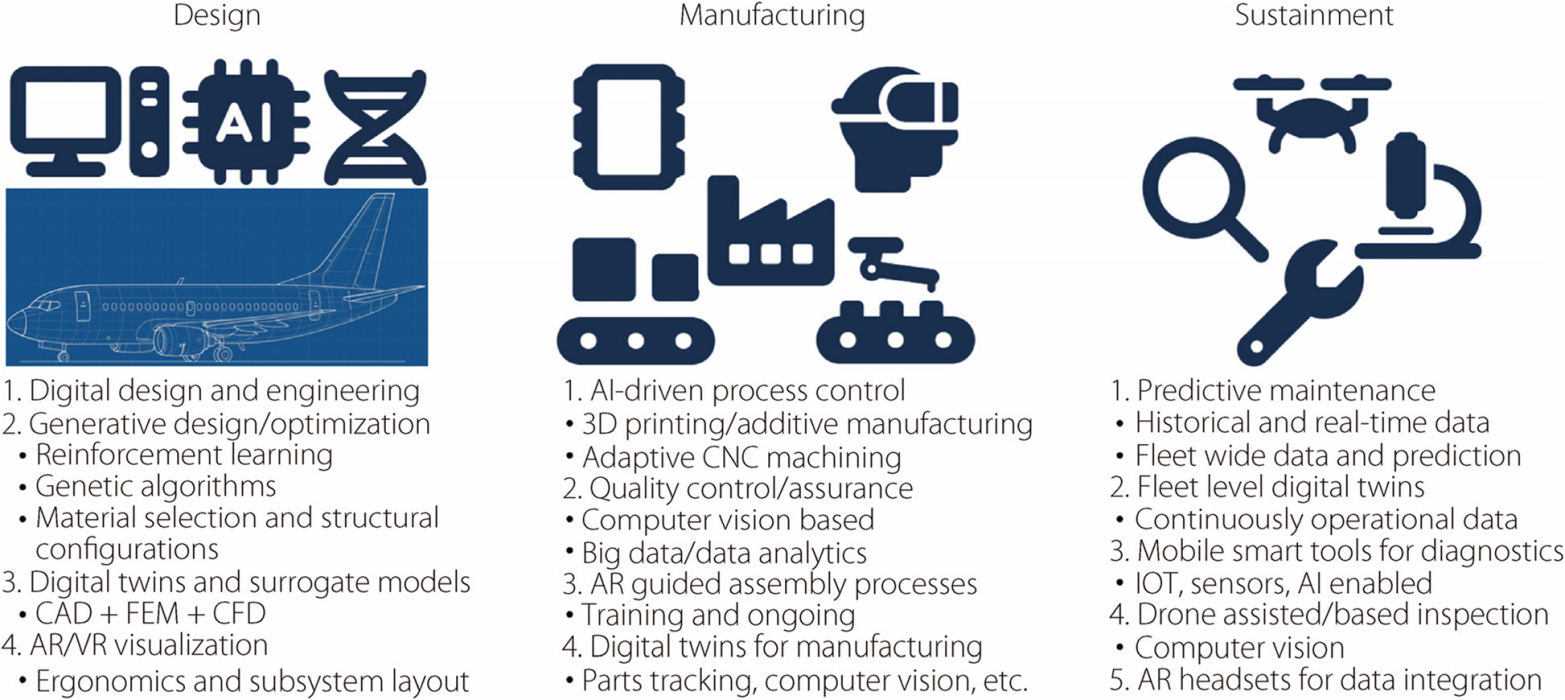
AI and mobile/immersive technologies across the aircraft engineering lifecycle

Digital engineering enables a connected, data-driven approach to the full lifecycle of aircraft and aerospace systems. AI and mobile computing are key enablers, supporting model-based design, smart manufacturing, and predictive maintenance. These technologies enhance efficiency, adaptability, and decision-making from concept development through to long-term sustainment. The integration of these technologies across the engineering lifecycle is illustrated in Fig. 9, highlighting their roles in design, manufacturing, and sustainment.

### Design

In aerospace engineering, the integration of advanced computational technologies such as digital twins, surrogate models, AI-driven simulations, generative AI, and real-time data analytics significantly enhances the design processes of airframes, engines, and aircraft systems. Central to these innovations is the concept of digital twins, which serves as a virtual counterpart to physical aircraft, enabling real-time monitoring and simulation of operational performance. By employing digital twins, engineers can predict outcomes, anticipate maintenance needs, and optimize performance parameters throughout the aircraft lifecycle [144, 145].

Digital twins are particularly effective for understanding the interactions between various aircraft components, such as airframes and propulsion systems. The representation of these interactions through holistic models allows for a more comprehensive analysis of how design changes influence the overall performance metrics, such as structural integrity, weight distribution, and aerodynamic efficiency [146, 147]. Notably, studies have highlighted that using a health and usage monitoring system with diagnostic algorithms can help detect issues early and improve the reliability of aircraft parts, such as landing gears [144]. This approach reduces maintenance costs and increases safety and availability by predicting problems before they occur.

Surrogate models complement digital twins by providing computationally efficient alternatives to high-fidelity simulations. These models are pivotal in scenarios where traditional computational fluid dynamics simulations become infeasible owing to their complexity and resource demands, particularly during the initial design iterations [148, 149]. For instance, an online learning model for predicting the output power of solar arrays on stratospheric airships demonstrated high accuracy and efficiency, highlighting the potential of surrogate models in aerospace engineering [150].

AI-driven simulations further enhance design processes by incorporating advanced algorithms capable of learning from past performances and predicting future outcomes. These simulations are adept at recognizing patterns in large datasets generated by physical tests and digital models. By leveraging ML techniques, AI can facilitate informed decision-making in the design process by predicting the effects of design modifications on performance without the need for extensive experimental validation. Recent advancements in AI, particularly DL, have been utilized to predict gas turbine temperatures and identify potential failures in aircraft systems, thus enabling a proactive approach to maintenance and safety management [151, 152].

### Manufacturing

Real-time data analytics underpin Industry 4.0 in aerospace, where data collected from various sensors and devices across manufacturing processes are analyzed instantaneously. This capability is important for monitoring operational parameters and identifying discrepancies in production, allowing manufacturers to take proactive measures to address potential defects before they become significant issues. For instance, integrated sensor networks in assembly lines facilitate continuous monitoring of equipment health and operational states, thereby supporting predictive maintenance strategies. Predictive maintenance utilizes AI algorithms to analyze historical data and predict the remaining useful life of components, which reduces downtime and maintenance costs while enhancing operational reliability [153].

AI algorithms play an important role in analyzing complex datasets collected during manufacturing processes. These algorithms, including ML models, enable the extraction of meaningful patterns from large volumes of data and the identification of correlations and trends that may not be apparent using traditional analysis methods. For instance, AI can be applied to optimize production schedules by analyzing forecasting models that consider historical performance and future demand. Such optimization minimizes lead times and maximizes production efficiency in the manufacture of aircraft components. Additionally, the adaptability of AI algorithms allows them to recalibrate themselves as new data streams, ensuring that predictions remain accurate and relevant [154].

In manufacturing, the integration of AI-driven solutions with digital twins significantly enhances data-driven decision-making. This technology allows the simulation of various scenarios, providing insights into the effect of adjustments in design or operation on the performance before implementing changes in a real environment. Consequently, manufacturers can optimize their processes without the risks and costs associated with physical trials. The effective utilization of digital twins, facilitated by AI algorithms, aids in improving product quality and streamlining operations [155].

### Sustainment

Another application of AI in aviation is predictive maintenance, which enables proactive responses to equipment degradation and failure. Aircraft maintenance is typically performed at scheduled intervals. However, this approach may not detect issues that develop between inspections. Predictive maintenance, powered by AI and ML, analyzes data from thousands of sensors across the aircraft to predict issues before they escalate [156]. This method not only reduces downtime and operational costs but also addresses potential safety risks by ensuring that all parts function optimally.

For instance, Delta Air Lines uses predictive maintenance technology to monitor its fleet in real time, which allows it to detect early warning signs of equipment failure [157]. By analyzing the patterns and anomalies in sensor data, airlines can minimize unexpected malfunctions and enhance PAX safety [158]. Another example is Airbus’s Skywise platform, which consolidates data from multiple sources to predict when parts may need repair or replacement, effectively preventing failures before they affect operations [159].

Compared with reactive maintenance approaches, predictive maintenance exemplifies a shift toward safety-driven asset management in aviation. This approach ensures that airlines can effectively allocate maintenance resources and reduce both financial and safety risks associated with unexpected breakdowns.

### Summary and case study

Table 5 provides an overview of the key findings of recent research on the integration of AI into aviation engineering. It highlights the main areas of focus, including design, development, manufacturing, and sustainment. The table lists the key points, technologies and methods used, and the impact or findings for each section, offering a summary of the utilization of AI to enhance the efficiency, safety, and effectiveness of airframes, engines, avionics, and other associated systems.

**Table 5:** Summary of AI for aviation engineering

Section	Key point	Technology/method	Impact/finding
Design	Optimization of aircraft design High-fidelity simulations Iterative design processes	Digital twins Surrogate models AI-driven simulations	Improved product lifecycle management Enhanced design efficiency and performance Reduced development time and costs
Manufacturing	Continuous monitoring of aircraft systems Quality control processes	Real-time data analytics AI algorithms	Timely interventions and reduced failures Improved efficiency and precision in quality control
Sustainment	Predictive maintenance Autonomous performance monitoring Documentation generation	AI and ML Generative AI Real-time data analytics	Reduced downtime and operational costs Optimized maintenance schedules Enhanced safety and efficiency

An example of this is highlighted by Singapore Airlines Engineering Company enhancing its productivity by integrating advanced robotics into its aircraft engine inspection processes [160]. The newly implemented robotic arm captures an average of 150 photographs per inspection, thereby accessing engine areas that are typically challenging for human technicians. This technology not only speeds up the inspection process but also improves accuracy using AI to identify discrepancies in engine components.

## Drones

### Overview of mobile AI applications in unmanned aerial systems

AI integration in drones has advanced autonomous navigation and decision-making, expanding applications in agriculture, surveillance, disaster response, and defense [161–163]. AI-driven drones use sensors to make real-time decisions, adapting to dynamic scenarios and optimizing flight paths [162–164]. Recent studies have addressed unmanned aerial system limitations with lightweight AI solutions. Fouda et al. [165] proposed a hierarchical AI framework for forest fire detection. Martinez-Alpiste et al. [166] developed a smartphone-based unmanned aerial system for object recognition. Alshanbari et al. [167] implemented an AI-powered target detection and payload release. McEnroe et al. [168] highlighted edge computing and AI convergence for unmanned aerial systems, thereby enhancing capabilities within resource constraints.

AI is enhancing unmanned aerial system operations in real-time decision-making for environmental scanning, path planning, and threat detection [169, 170]. AI-enabled unmanned aerial systems use advanced sensors and ML for autonomous navigation in complex environments, optimizing trajectories using heuristic A* algorithms and simultaneous localization and mapping (SLAM) techniques [170]. AI improves navigation and decision-making using DNNs and RL [162]. Emerging trends include federated learning (FL) and distributed ML for unmanned aerial systems [171]. Adaptive mission planning for unmanned aerial systems dynamically updates navigation based on environmental feedback. These methods include fuzzy Kalman filtering, Bayesian networks, and evolutionary algorithm-based optimization [172–174]. These approaches improve the mission success and adaptability to hazards. Advancements in AI and ML, such as ANNs and SVMs, have enhanced unmanned aerial systems navigation, particularly for small unmanned aerial systems [175]. Challenges remain in ensuring predictable system behavior for certification and uncrewed ATC requirements. AI-driven resource allocation and payload management optimize unmanned aerial system operations in multi-mission scenarios. AI algorithms enable real-time decisions and optimize mission requirements within hardware constraints [171]. Studies have demonstrated AI-powered unmanned aerial systems for autonomous payload transport using target detection and 3D-printed release mechanisms [167]. Advanced frameworks integrate object detection and decision-making for precise payload delivery [176]. Resource allocation and cooperative path planning minimize flight time and enhance situational awareness and operational effectiveness for various applications [177].

**Fig. 10:**
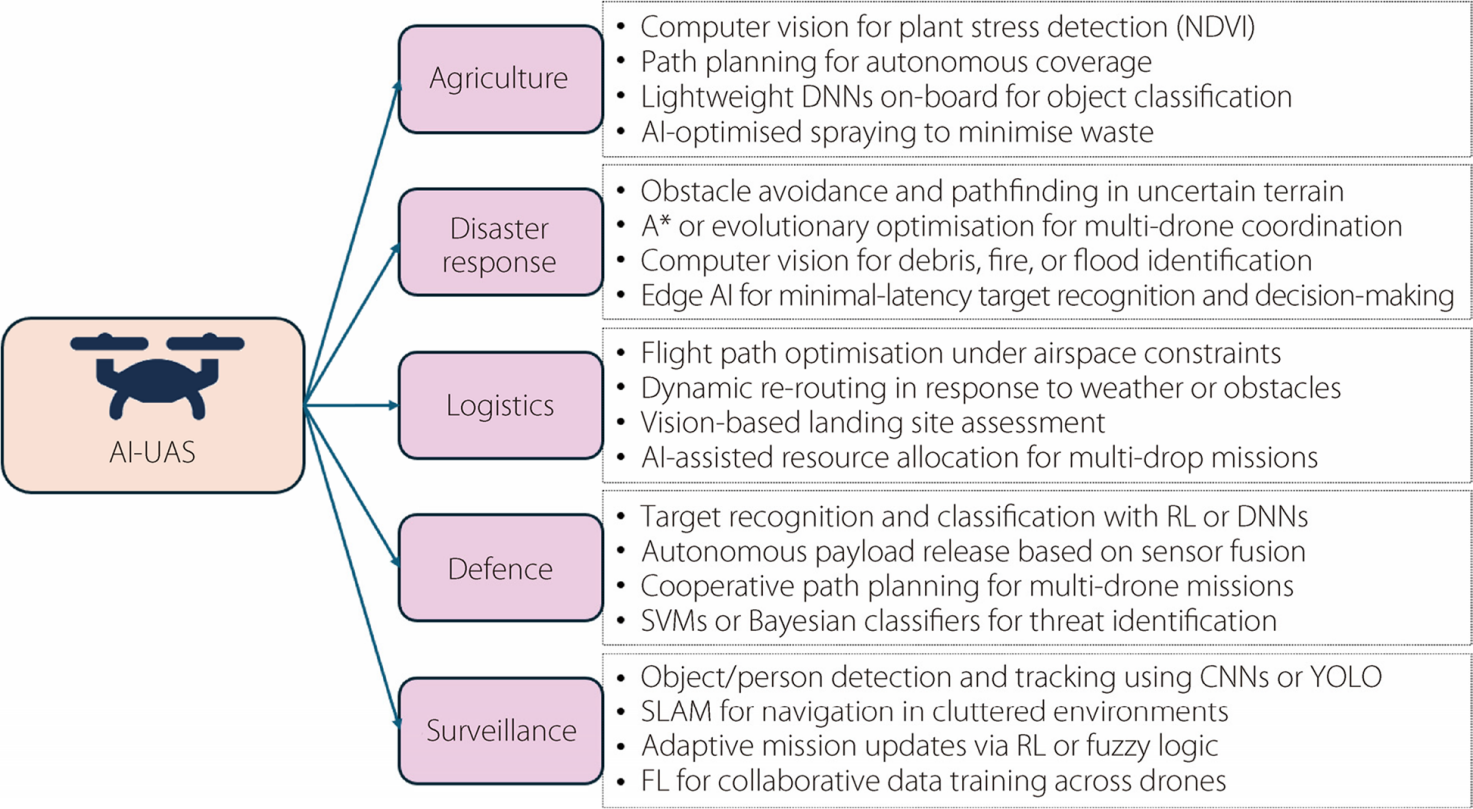
Application domains of AI-enabled unmanned aerial systems and their associated core functions

AI-enabled unmanned aerial system supports a wide range of operational domains, each with distinct functional requirements and computational constraints. These are illustrated in Fig. 10, highlighting the key applications and associated AI capabilities across agriculture, surveillance, disaster response, defence, and logistics.

### AI models and hardware

TinyML enables DL on low-power, resource-constrained devices by combining IoT and edge computing for efficient ML [178–180]. Recent advancements, such as TinyVQA, have demonstrated high accuracy and low latency in visual question answering tasks on TinyML hardware, paving the way for edge-level AI applications [181, 182]. Research on integrating edge computing, AI, and FL in unmanned aerial system networks has addressed latency, privacy, and efficiency challenges. Edge AI enhances unmanned aerial system performance by processing data closer to the source, whereas FL allows distributed learning without sharing raw data, thereby improving privacy and reducing communication overhead [168, 183–185].

Weight and power constraints affect the AI hardware choices for drones. Although graphics processing units (GPUs) are powerful, their high-power consumption is a drawback. Field-programmable gate arrays (FPGAs) offer energy-efficient alternatives; however, added weight may be an issue. Researchers have explored low-precision quantization and model compression to optimize AI-enabled drones [186–190]. Advancements in the battery and thermal management of lightweight drones have focused on the efficiency and prevention of shutoffs. These innovations include passive thermal management systems and AI-enhanced battery management systems (BMSs) for accurate performance prediction. Edge AI ensures low latency and energy consumption for unmanned aerial system-based IoT services [191–193].

### Real-time navigation and safety

Visual-inertial navigation systems for unmanned aerial systems integrate computer vision with inertial measurement units and global positioning system (GPS) to enhance navigation accuracy, particularly in GPS-denied environments [194, 195]. SLAM-based methods and Kalman filters are commonly used for sensor fusion, providing redundant navigation solutions and continuous movement estimation even during GPS blockages [196, 197]. This multi-sensor approach offers greater accuracy and update rates than individual sensors [195]. Research on lightweight computer vision and light detections and ranging (LiDAR) based navigation methods has enabled autonomous navigation in challenging environments. Vision-aided systems with day and thermal cameras support multi-uncrewed aerial vehicle (UAV) flocking without wireless communication, whereas omni-directional stereo vision and LiDAR sensors facilitate obstacle perception and localization [198–201]. These advances enable autonomous navigation, mapping, and obstacle avoidance in GPS-denied settings. AI approaches for path optimization and collision avoidance in unmanned aerial systems include real-time 3D path planners that use heuristic A* algorithms and RL-based decentralized collision avoidance [170, 202]. Combining RL with optimization theory improves the trajectory planning and performance in UAV communication networks [203]. RL-based navigation outperforms traditional AI algorithms and enhances unmanned aerial system capabilities in dynamic environments [204].

DL and computer vision techniques enable real-time obstacle detection and avoidance in complex unmanned aerial system environments including GPS-denied zones and crop environments [205]. Recent advancements have integrated monocular and binocular vision systems with DL for obstacle detection and spatial localization using GPU-powered embedded devices for high-level autonomy [206, 207]. Challenges remain in developing robust real-time systems, owing to unmanned aerial system autonomy and weight limitations [208]. Researchers are creating custom datasets and advanced neural network architectures to improve the accuracy and inference speed, thereby enhancing unmanned aerial system operations in precision agriculture and IoT deployments [207].

Research on autonomous emergency landing systems for unmanned aerial systems in urban environments has employed geometric flight planning, AI-based landing zone identification, and machine vision algorithms for real-time site assessment [209–212]. Fail-safe navigation protocols use nontraditional data sources to identify safe landing sites, thereby improving safety and reliability in urban settings [213–216].

AI models for detecting anomalies in unmanned aerial systems enhance safety and reliability. Approaches include long short-term memory (LSTM) AE models for structural anomaly detection, recursive least squares for actuator failure detection, and DL-based log analysis for anomaly detection in drone logs [217–220]. Multimodal regression models using a relevance vector machine adapt to different flight modes, thereby improving adaptability to anomaly detection [221]. These AI-driven approaches enhance UAV safety through predictive maintenance and in-flight corrective measures [222, 223].

### Mission adaptability and autonomous decision-making

AI-enabled adaptive mission updates for unmanned aerial systems will optimize multi-objective intelligence, surveillance, and reconnaissance (ISR) missions by considering weather, new targets, and airspace restrictions [174]. Adaptive perception systems select appropriate algorithms based on the environmental conditions will improve their performance [224]. Multi-unmanned aerial system coordination systems and integrated planning update wind models for safe navigation in complex weather will be possible [225, 226]. These approaches enhance the adaptability of unmanned aerial systems for surveillance missions through real-time feedback.

AI enhances swarming and collaborative behavior among drones, enabling autonomous navigation, object recognition, and environmental monitoring [163]. AI algorithms facilitate swarm formation, task allocation, and decision-making, thereby improving UAV network performance [227, 228]. Behavior-based AI paradigms optimize search efficiency through role reallocation based on the swarm size [229]. These advancements have expanded the applications of AI-driven drones across various industries [230].

AI-equipped unmanned aerial systems are increasingly being used in search and rescue missions, offering real-time monitoring and human detection [231–233]. AI improves path planning, coordination, and adaptability during critical missions. In 5G networks, system and edge intelligence enhance UAV mobility and connectivity, thereby improving search and rescue efficiency through real-time image processing and rapid information transfer [234].

### Summary and case study

Table 6 provides an overview of the key findings of recent studies on the integration of AI into drone operations. The table outlines the key points, technologies and methods used, and the impact or findings for each section, offering a summary of the utilization of AI to enhance the efficiency, safety, and adaptability of drones.

**Table 6:** Summary of AI for drones

Section	Key point	Technology/method	Impact/finding
Overview of mobile AI applications in unmanned aerial system	AI integration in drones for autonomous navigation and decision-making Expanded applications in agriculture, surveillance, disaster response, and defense Lightweight AI solutions for unmanned aerial system limitations	AI-driven sensors ML DL RL Hierarchical AI framework Smartphone-based unmanned aerial system Edge computing	Enhanced real-time decision-making Optimized flight paths Improved unmanned aerial system capabilities within resource constraints
AI models and hardware	TinyML for low-power devices Edge computing and FL Weight and power constraints on AI hardware Battery and thermal management	TinyML Edge AI FL GPUs, FPGAs Low-precision quantization Model compression AI-enhanced BMSs	High accuracy and low latency in AI tasks Improved unmanned aerial system performance Enhanced privacy and reduced communication overhead Efficient and reliable AI-enabled drones
Real-time navigation and safety	Visual-inertial navigation systems Lightweight computer vision and LiDAR-based navigation AI for path optimization and collision avoidance DL and computer vision for obstacle detection Autonomous emergency landing systems AI models for anomaly detection Real-time decision-making for environmental scanning, path planning, and threat detection	SLAM-based methods Kalman filters Heuristic A* algorithms RL Monocular and binocular vision Geometric flight planning LSTM AE models Recursive least squares DL-based log analysis Advanced sensors ML Fuzzy Kalman filtering Bayesian networks Evolutionary algorithm-based optimization	Enhanced navigation accuracy Autonomous navigation in challenging environments Improved trajectory planning and performance Efficient obstacle detection and avoidance Safe and reliable emergency landings Enhanced UAV safety through predictive maintenance Improved mission success and adaptability to hazards
Mission adaptability and autonomous decision-making	Adaptive mission updates for unmanned aerial system surveillance AI for swarming and collaborative behaviors AI in search and rescue missions AI-driven resource allocation and payload management	Multi-objective ISR missions Adaptive perception systems Multi-unmanned aerial system coordination AI algorithms for swarm formation System intelligence and edge intelligence in 5G networks Target detection 3D-printed release mechanisms Object detection and decision-making Cooperative path planning	Improved unmanned aerial system adaptability in surveillance Enhanced autonomous navigation and object recognition Efficient path planning and coordination Improved search and rescue efficiency Optimized mission requirements within hardware constraints Enhanced situational awareness and operational effectiveness

Recently, AI-enabled drones have been deployed to enhance disaster response efforts by providing real-time object recognition and situational awareness [235]. These drones, equipped with advanced sensors and mobile edge computing, were used in the Valdgale district of Latvia to identify areas with the highest risk of fire reignition. Rapid information collection and analysis have significantly improved the efficiency and effectiveness of response efforts, exhibiting the potential of AI and mobile computing in transforming disaster response operations.

## Discussion and conclusions

### Current applications of AI and immersive technologies in aviation

The reviewed sources highlighted the wide range of applications of AI and immersive technologies across diverse aviation sectors. In education and training, AI is transforming pilot assessment strategies by providing personalized feedback and enabling faster skill development. AI-powered flight simulators offer a safe and controlled environment for practicing complex scenarios and improving decision-making and response times. Beyond pilot training, AI enhances aviation safety in several ways, as follows:

- Pilot assistance systems: AI systems analyze flight data and provide real-time recommendations, aiding pilots in challenging situations and reducing cognitive loads.
- Error mitigation: AI identifies common risk factors, aiding in the development of targeted training programs to address human errors.
- SMS: AI streamlines risk assessment and the identification of safety trends, thereby facilitating proactive safety measures.
- Accident analysis: AI helps identify the root causes of incidents by processing vast amounts of data, enabling the continuous refinement of safety protocols.

The sources in this review also detail the significant impact of AI on airport operations. For instance, AI-powered systems can optimize baggage handling, thereby reducing lost luggage and improving reclamation efficiency. AI also optimizes ground vehicle routing and allocation, thereby minimizing delays and fuel consumption. AI combined with wearable technology can enhance worker health and safety, particularly in physically demanding tasks, such as baggage handling. AI-enabled systems can automate FOD detection, thereby improving the safety and efficiency of airfields. Finally, AI plays a crucial role in ATM, optimizing aircraft trajectories and enhancing traffic flow management, thereby contributing to smoother and safer air travel.

### Lightweight AI models and hardware for mobile applications

The reviewed sources emphasize the importance of lightweight AI solutions, particularly for resource-constrained environments such as drones. TinyML, an emerging field that focuses on the DL of embedded devices with low power consumption, is particularly relevant for drone applications. The sources also discussed various hardware considerations for mobile AI in drones, including the trade-offs between the use of energy-efficient FPGAs, power-hungry GPUs, or custom platforms with hardware accelerators. Battery and thermal management are also crucial aspects, with research focusing on improving the efficiency and preventing unexpected shutdowns. All of these are significant for mobile computing related to AR, which will become widespread across the aviation industry.

### Benefits and challenges of AI integration

This review illustrates the numerous benefits of integrating AI and immersive technologies in aviation, including enhanced safety, improved efficiency and productivity, reduced environmental impact, and cost savings. However, some challenges were also noted. Ethical considerations and regulatory frameworks for AI in safety-critical decisions were addressed. Addressing algorithmic bias, ensuring cybersecurity, and managing the relationship between human operators and AI systems are crucial.

#### Algorithm bias

The aviation industry has integrated AI and ML technologies, introducing opportunities and challenges such as algorithmic bias [4, 236]. Algorithmic biases and systematic errors in AI decision-making can arise from skewed training data or incomplete scenarios [237, 238]. In aviation, even slight biases can affect risk assessments, maintenance predictions, and operational reliability [4, 236, 239]. These errors may undermine safety regulations and protocols [4, 236].

Algorithmic bias stems from imbalanced datasets and incomplete records [237]. AI models may perform well under common conditions but poorly in critical scenarios if the data are not comprehensive [239]. Bias is problematic when data from rare events are underrepresented, leading to incorrect predictions [237]. Historical datasets reflecting past practices can perpetuate outdated methods in AI algorithms [238]. Rigorous data curation and preprocessing are required to ensure a comprehensive representation before model training [237].

Algorithmic bias in aviation is significant because the industry relies on high operational reliability and safety [4, 236]. AI systems for flight control, route planning, and maintenance scheduling operate in environments where errors can have severe consequences [239]. Misclassified sensor readings or misinterpreted anomalies can compromise flight safety and result in losses [239]. Biases may alter risk assessments and performance predictions integral to operations and emergency protocols [239].

Safety in aviation is non-negotiable, and AI enhances predictive safety measures [4, 236]. However, biased AI models can degrade the ability to predict failures, leading to a false sense of security [239]. Undetected biases disrupt operational efficiency and increase risk during peak traffic periods [4, 236].

AI in predictive maintenance promises timely failure detection and reduces downtime [240, 241]. Biased models with flawed training data can misestimate breakdown likelihoods, resulting in unnecessary maintenance or dangerous oversight [239, 240]. This threatens the operational efficiency, PAX safety, and aircraft reliability [241]. Systematic biases in maintenance systems can misallocate resources, posing risks to regulated environments [237, 240]. Continuous monitoring and recalibration of maintenance AI systems are essential for minimizing the impact of bias on aviation safety [4, 236].

AI systems for fault detection and health monitoring in maintenance are vulnerable to bias, owing to their reliance on historical data [240, 241]. Incomplete or skewed maintenance records can lead to misdiagnoses, resulting in unnecessary costs or overlooking signs of failure [239, 240]. Accurate predictive systems influence maintenance schedules and resource allocation essential for aircraft safety [4, 236, 241]. Ongoing bias mitigation and regular recalibration are crucial for a reliable operation [242].

Training data for aviation AI models are often obtained from historical records that can include biases or fail to capture emerging trends [237, 238]. Without updating or cross-validation, AI models may embed outdated practices, leading to misaligned decisions [239, 241]. This poses risks where modern operations must adapt to unprecedented situations [237, 242]. Excessive reliance on historical data without proper checks weakens the ability of the model to generalize and accurately forecast anomalies or failures [237, 239]. Evolving datasets and proactive bias detection are crucial for reliable aviation AI systems [243].

Certification challenges for AI-based systems highlight the need to demonstrate unbiased operational behavior before approval [241, 244]. Regulatory demands require rigorous evidence of safe AI performance with bias-threatening certifications [4, 236, 241]. Robust bias detection and mitigation strategies are required for the development of AI [244, 245]. Certification processes ensure that AI systems satisfy aviation safety standards [241]. Collaboration among developers, operators, and regulators is essential to align AI performance with safety and fairness directives [214, 239].

#### Cybersecurity

AI evolution in aviation, while promising, also has significant implications for cybersecurity, both advantageous and challenging [246]. The strategic integration of human resources alongside AI systems has been identified as essential to ensure that these technologies complement established cybersecurity strategies, thereby reducing time-to-detect and allowing for quicker responses to cyber incidents [247].

The same AI systems that enhance threat analysis in aviation have widened the potential attack surface. The complexity introduced by integrating AI with critical systems can be exploited by adversaries through adversarial attacks and data poisoning, which may lead to erroneous outputs from otherwise trusted ML models [248]. The vulnerability of interconnected systems to such sophisticated exploits is of particular concern in aviation, where communication interfaces and sensor networks are paramount for safety [249]. As the digital and physical domains merge in this sector, any compromise in AI system integrity could lead to cascading failures in both operational and cybersecurity defenses.

The deployment of AI in aviation cybersecurity requires a new framework for regulatory oversight and risk management. A growing consensus has emerged that the existing global cybersecurity policies are insufficient to address the complexities introduced by AI [250]. New policies must be devised to keep pace with technological innovations while effectively addressing challenges, such as the ‘black-box’ nature of numerous AI systems. This opacity may hinder transparency and erode trust, thereby necessitating approaches that include XAI and human oversight to ensure that critical decisions are interpretable and accountable [251].

#### Human machine interactions

One significant implication is that the role of AI in aviation enhances operational decision-making by processing vast datasets and supporting real-time responses. However, this benefit depends on the alignment of human expertise with AI outputs. The effective deployment of AI in aviation requires collaboration, wherein human insight complements machine efficiency [252]. Research indicates that leadership and human resource practices must evolve concurrently with technology to optimize performance and safety [4]. Such an evolution is critical because AI systems, although adept at rapid data analysis and anomaly detection, still rely on human judgment and oversight to contextualize outcomes and make final decisions.

In addition, human operators must understand and interpret AI decisions in complex, safety-critical scenarios. The safety-critical nature of aviation requires AI systems to be interpretable to build trust between operators and automated systems [253]. Moreover, effective design strategies advocate for the visualization of AI decision-making outputs in ways that enhance operator confidence [254]. When operators comprehend the logic behind AI recommendations, they are better positioned to manage situations in which reliance on artificial judgment is necessary yet not absolute. This transparency can help reduce cognitive bias and automated complacency, which can compromise safety.

As AI systems are integrated into aviation workflows, the variability in operator skill sets and cognitive styles becomes critical. Papanikou et al. [255] illustrated significant variability among operators in their ability to interact with advanced systems, emphasizing the need for tailored training programs that address not only technical skills but also stress management. In this context, training tools developed using AI offer the potential for adaptive learning environments that adjust to the evolving needs of operators while reinforcing critical safety protocols [256].

### Future directions and potential

This study points toward a future in which lightweight, mobile AI, and immersive technologies will continue to be incorporated into the aviation industry. The ongoing development of more sophisticated algorithms, combined with advancements in hardware and sensor technologies, will lead to even more capable and autonomous systems. The increasing integration of AI with AR and VR will create new training and operational paradigms, further enhancing safety, efficiency, and human-machine collaboration. For instance, future research could explore how AI can be combined with AR for real-time guidance during maintenance tasks and how these technologies can create immersive training simulations for ground crews and maintenance technicians. AI will continue to play a pivotal role in optimizing complex operations ranging from ground handling and ATC to predictive maintenance and accident prevention. Future directions and potentials are shown in Fig. 2.

#### Aviation safety

AI will be important for enhancing safety in the aviation industry by providing advanced tools for monitoring and decision-making. AI systems can analyze vast amounts of data in real time to detect anomalies, predict potential failures, and recommend preventive actions, thereby reducing the risk of accidents [257]. One potential application of mobile computing in this context is eye-tracking technology, which can monitor pilots’ attention and fatigue levels. Using AI to analyze eye movement patterns, the system can alert pilots and ground controls to signs of fatigue or distraction, thereby ensuring that timely interventions are made to maintain high safety standards [258].

#### Aviation maintenance

Research on predictive maintenance has demonstrated that the integration of AI with IoT sensors and big data analytics leads to significant improvements in aircraft health monitoring and maintenance scheduling. Kabashkin and Perekrestov [259] presented an ecosystem that synergizes IoT and AI to transition from basic health monitoring to a comprehensive health management system. Complementing this, Wang et al. [260] proposed an interpretable ML framework that not only improved prediction accuracy but also translated directly into operational cost savings. Korba et al. [261] detailed how an algorithmic approach that aggregates real-time aircraft and maintenance data can enable advanced predictive analytics.

#### ATC

ATC systems undergo similar digital transformations. Research has highlighted the potential role of intelligent assistants in cockpits and ATC environments [240]. Such systems could eventually lead to more autonomous operations, thereby reducing the human workload during peak traffic hours. Research on the impacts of external stressors, such as the COVID 19 pandemic, on ATC proposed that AI‐enabled training simulations could help prepare controllers for rapid operational scale up post disruptions [262]. Hurter et al. [263] studied ML in ATC highlighting that advanced predictive models and data‐driven decision support systems are essential for managing increasing air traffic densities.

#### Ground handling

In ground handling, the advent of mobile computing and digital twin technologies has advanced the state-of-the-art technology. When integrated with IoT and AI, mobile platforms can support real-time coordination among ground crews-optimizing processes such as refueling, baggage handling, and routine inspections. Yiu et al. [264] described a digital twin‐based platform that creates virtual counterparts of flight and ATC operations; such technology can readily be extended to model and optimize ground handling logistics. This convergence of mobile computing with AI not only streamlines resource allocation but also reduces operational delays by ensuring timely and accurate data exchange across various ground operations [265]. These systems facilitate real-time coordination among disparate operational units, reducing delays and improving the safety and efficiency of cargo transfer and baggage delivery processes [266].

#### Aviation training and education

Aviation training simulations are undergoing a paradigm shift with the application of AR, VR, and other immersive technologies. Moruzzi et al. [267] provided a concrete example of the implementation of AR interfaces in remote and virtual control towers to enhance situational awareness and decision‐making among air traffic controllers. Ziakkas et al. [268] further discussed the challenges and potential of immersive technologies, noting that realistic simulation environments offer pilot and controller training that mirrors the actual operational conditions more closely than traditional methods.

#### Crew

AI improves crew scheduling in aviation by addressing issues such as pilot fatigue and optimizing complex scheduling processes. Recent advancements in AI-based fatigue recognition have significantly enhanced aviation safety by identifying and warning of potential incidents caused by impaired cognition among aviation professionals [15]. Additionally, AI tools are transforming the traditional manual and time-consuming scheduling process for air force missions and training. By incorporating neural networks, RL, and linear programming, these AI-enabled tools generate optimal schedules that improve unit readiness and reduce disruptions to missions, training, and aircrew personal life [269].

#### PAXs

AI also enhances the PAX experience in aviation by increasing efficiency and personalizing services. Research has highlighted how AI applications, including ML, DL, RL, and NLP, could improve predictive analytics, resource optimization, safety, autonomous processes, and PAX experiences [270]. Additionally, the targeted and controlled information provided by modern technologies at airports plays a crucial role in reducing PAX stress and enhancing their journeys. Direct communication channels and personalized updates help create a seamless and enjoyable experience from arrival to departure [271].

#### Airlines

AI not only improves aspects of PAX but also provides corporate benefits. For instance, dynamic pricing is crucial for airline revenue management, which requires quick adaptation to market changes and customer behavior. Advanced AI algorithms have been developed to handle the complex pricing of multiple flights, outperforming traditional methods by quickly learning and adapting to market conditions [272]. Additionally, a new pricing approach with patient customers suggests alternating high and low prices to increase revenue, particularly when customers are willing to wait for better deals [273]. These and other AI-driven methods enhance airlines’ effectiveness and efficiency.

#### Operations

In the realm of flight operations and navigation, increasing congestion in airspaces has prompted the aviation community to seek technologies for efficiently managing integrated multi-aviation airspaces. Solutions are being explored to unify air navigation regulations, enabling diverse aviation sectors to coexist in a shared airspace, and technologies will facilitate the integration of different aviation capabilities through software engineering and AI traits such as knowledge representation [274]. This is even more significant for UAM, where AI has been proposed to manage dense low-altitude airspaces effectively [275].

#### Sustainable aviation

Aviation technology is another important area of research and development. Research suggests that future engineering design efforts should focus on utilizing sustainable materials and optimizing structural designs by leveraging AI and advanced numerical modeling to improve structural performance and safety [276]. The area with the greatest gain from these technological advancements in aviation is UAM, which relies heavily on AI for future fully autonomous operations [1].

#### Airport security

Airport security is experiencing significant advancements driven by advanced technologies. AI-based surveillance systems and pattern recognition algorithms are increasingly utilized to preemptively identify threats and automate routine security tasks, thereby enhancing overall airport resilience [277]. In addition, mobile computing devices equipped with AI can assist security personnel by providing real-time data analysis and alerts, thereby improving response times and coordination during security incidents [278].

### Conclusions

AI and immersive technologies are transforming the aviation industry and offer significant benefits in terms of safety, efficiency, and sustainability. Although challenges remain, continuous advancements in these fields promise a future in which aviation will be safer, more efficient, and more environmentally friendly. Continued research and collaboration among industry stakeholders, researchers, and regulatory bodies are crucial for harnessing the full potential of these transformative technologies.

## Data Availability

The authors confirm that the data supporting the findings of this study are available within the article.

## Abbreviations

PAX: Passenger
AI: Artificial intelligence
FAA: Federal Aviation Administration
MRO: Maintenance, repair, and overhaul
ATC: Air traffic control
AR: Augmented reality
VR: Virtual reality
ML: Machine learning
ANN: Artificial neural network
SVM: Support vector machine
DL: Deep learning
HF: Human factor
ATM: Air traffic management
UAS: Uncrewed aerial system
RL: Reinforcement learning
AE: Autoencoder
DNN: Deep neural network
ATTOL: Airbus’s Autonomous Taxi, Takeoff, and Landing
xR: Extended reality
IoT: Internet of Things
NLP: Natural language processing
RFID: Radio frequency identification
3D: Three-dimensional
MILP: Mixed-integer linear programming
FOD: Foreign object debris
SESAR: Single European Sky ATM Research
XAI: EXplainable artificial intelligence
SLAM: Simultaneous localization and mapping
GPU: Graphics processing unit
FPGA: Field-programmable gate array
BMS: Battery management system
GPS: Global positioning system
LiDAR: Light detections and ranging
UAV: Uncrewed aerial vehicle
LSTM: Long short-term memory
ISR: Intelligence, surveillance, and reconnaissance
UAM: Urban air mobility
SMS: Safety management system
FL: Federated learning
